# Cardiac Troponin T and Troponin I in the General Population: Comparing and Contrasting Their Genetic Determinants and Associations With Outcomes

**DOI:** 10.1161/CIRCULATIONAHA.118.038529

**Published:** 2019-04-24

**Authors:** Paul Welsh, David Preiss, Caroline Hayward, Anoop S.V. Shah, David McAllister, Andrew Briggs, Charles Boachie, Alex McConnachie, Sandosh Padmanabhan, Claire Welsh, Mark Woodward, Archie Campbell, David Porteous, Nicholas L. Mills, Naveed Sattar

**Affiliations:** 1 Institute of Cardiovascular and Medical Sciences (P.W., S.P., C.W., N.S.), University of Glasgow, United Kingdom.; 2 Institute of Health and Wellbeing (A.B.), University of Glasgow, United Kingdom.; 3 Robertson Centre for Biostatistics (C.B., A.M.), University of Glasgow, United Kingdom.; 4 MRC Population Health Research Unit, Clinical Trial Service Unit and Epidemiological Studies Unit (D. Preiss), University of Oxford, United Kingdom.; 5 The George Institute for Global Health (M.W.), University of Oxford, United Kingdom.; 6 MRC Human Genetics Unit, MRC Institute of Genetics and Molecular Medicine (C.H.), University of Edinburgh, United Kingdom; 7 BHF Centre for Cardiovascular Science (A.S.V.S., N.L.M.), University of Edinburgh, United Kingdom; 8 Centre for Genomic and Experimental Medicine, MRC Institute of Genetics and Molecular Medicine (A.C., D. Porteous), University of Edinburgh, United Kingdom; 9 Usher Institute for Population Health Sciences and Informatics (A.C.), University of Edinburgh, United Kingdom; 10 The George Institute for Global Health, University of New South Wales, Sydney, Australia (M.W.).; 11 Department of Epidemiology, Johns Hopkins University, Baltimore, MD (M.W.).

**Keywords:** cardiovascular diseases, genetics, risk factors, troponin, troponin T

## Abstract

**Background:**

There is great interest in widening the use of high-sensitivity cardiac troponins for population cardiovascular disease (CVD) and heart failure screening. However, it is not clear whether cardiac troponin T (cTnT) and troponin I (cTnI) are equivalent measures of risk in this setting. We aimed to compare and contrast (1) the association of cTnT and cTnI with CVD and non-CVD outcomes, and (2) their determinants in a genome-wide association study.

**Methods:**

High-sensitivity cTnT and cTnI were measured in serum from 19 501 individuals in Generation Scotland Scottish Family Health Study. Median follow-up was 7.8 years (quartile 1 to quartile 3, 7.1–9.2). Associations of each troponin with a composite CVD outcome (1177 events), CVD death (n=266), non-CVD death (n=374), and heart failure (n=216) were determined by using Cox models. A genome-wide association study was conducted using a standard approach developed for the cohort.

**Results:**

Both cTnI and cTnT were strongly associated with CVD risk in unadjusted models. After adjusting for classical risk factors, the hazard ratio for a 1 SD increase in log transformed troponin was 1.24 (95% CI, 1.17–1.32) and 1.11 (1.04–1.19) for cTnI and cTnT, respectively; ratio of hazard ratios 1.12 (1.04–1.21). cTnI, but not cTnT, was associated with myocardial infarction and coronary heart disease. Both cTnI and cTnT had strong associations with CVD death and heart failure. By contrast, cTnT, but not cTnI, was associated with non-CVD death; ratio of hazard ratios 0.77 (0.67–0.88). We identified 5 loci (53 individual single-nucleotide polymorphisms) that had genome-wide significant associations with cTnI, and a different set of 4 loci (4 single-nucleotide polymorphisms) for cTnT.

**Conclusions:**

The upstream genetic causes of low-grade elevations in cTnI and cTnT appear distinct, and their associations with outcomes also differ. Elevations in cTnI are more strongly associated with some CVD outcomes, whereas cTnT is more strongly associated with the risk of non-CVD death. These findings help inform the selection of an optimal troponin assay for future clinical care and research in this setting.

## Abstract 2

Supplemental Digital Content is available in the text.

**Clinical Perspective**

**What Is New?**

- High-sensitivity cardiac troponin I and troponin T have different associations with health outcomes, including specific cardiovascular disease outcomes, in the general population.
- Upstream genetic causes of elevated cardiac troponin I and troponin T in healthy people also appear distinct from each other.

**What Are the Clinical Implications?**

- Cardiac troponin I appears to be a more specific marker of risk of composite cardiovascular disease and coronary heart disease, whereas cardiac troponin T is more strongly associated with risk of non–cardiovascular disease death.
- Both cardiac troponin I and cardiac troponin T were associated with heart failure and cardiovascular disease death.
- These findings help inform the selection of an optimal troponin assay for future clinical care and research in the general population.

The 99th centile of high-sensitivity cardiac troponin T (cTnT) and troponin I (cTnI), derived from a normal reference population, is used to detect myocardial necrosis as part of a diagnosis of myocardial infarction.^1,2^ However, low-grade elevations in troponin in the general population, well below the diagnostic threshold, are also associated with future cardiovascular disease (CVD) events and may have a role in screening the general population for CVD risk.^3,4^

In existing cohort studies, high-sensitivity cTnI or cTnT are frequently measured individually without an evidence-based decision as to which biomarker might be preferable to achieve a specific aim. This is reflected in a recent study-level meta-analysis where 13 studies measured cTnI and 7 studies measured cTnT, although none measured both.^4^ One study has compared the performance of different cTnI assays in the general population.^5^ Our own recent data highlight differential cross-sectional associations of cTnI and cTnT with classical CVD risk factors, and their relatively weak association with each other in a general population.^6^ Therefore, there is a need for a systematic approach to compare high-sensitivity cTnI and cTnT and their associations with a range of CVD and non-CVD health outcomes. Such a study has the potential to prioritize which of these assays should be used to improve CVD risk prediction, and for future measurement in established biobanks.

As a separate issue, the causal upstream determinants of troponin elevation in the general population are not clear. The use of genetic variants to investigate potential causal pathways is now well established.^7^ A genome-wide association study (GWAS) of 11 544 individuals has been reported for cTnT,^8^ but there are currently limited data reported on genetic determinants of either troponin. Contrasting the upstream genetic determinants of low-grade elevations in cTnI and cTnT would allow further mechanistic insight into potential differences in their causal determinants.

Using clinical high-sensitivity assays, we measured both cTnT and cTnI in 19 501 individuals in the Generation Scotland Scottish Family Health Study (GS:SFHS). We aimed to contrast the associations of cTnI and cTnT with risk of CVD and non-CVD health outcomes, and to conduct a GWAS for both of the biomarkers to investigate differences in both the causes and consequences of their elevation.

## Methods

The data, analytic methods, and study materials will be made available to other researchers for purposes of reproducing the results or replicating the procedure subject to a successful project application to the Generation Scotland Access Committee, and requisite associated ethical approval for access to linked health data from NHS Scotland.

### Study Recruitment

The recruitment and design of GS:SFHS has been reported elsewhere.^9,10^ During 2006 to 2010, potential participants (aged 35–65 years) were identified and invited at random from collaborating general medical practices in Scotland. Participants were asked to identify ≥1 first-degree relatives aged ≥18 years who would also be able to participate. Subsequently, 21 476 participants aged between 18 and 98 years attended a staffed research clinic in Scotland. Participants completed a health questionnaire, and had physical and clinical characteristics measured according to a standardized protocol.^10^ Past medical history, including a diagnosis of diabetes mellitus (type 1 or type 2) and CVD (previous myocardial infarction or stroke), and use of medications was self-reported. Family history of CVD was defined as a self-report of parents or siblings having heart disease or stroke. Fasting blood samples were taken, according to a standard operating procedure, and serum samples were separated. Baseline biochemistry including total cholesterol, high-density lipoprotein cholesterol, and serum creatinine, were measured at the time of collection, and additional serum aliquots were stored at –80°C for future biochemical analyses. Scottish Index of Multiple Deprivation scores, nationally compiled composite measures of small-area deprivation, were derived from participant postcodes.^11^ The study obtained written informed consent from all participants and received ethical approval from the National Health Service Tayside Committee on Medical Research Ethics (REC Reference Number: 05/S1401/89).

### Measurement of High-Sensitivity Troponins

High-sensitivity cTnT (Roche Diagnostics) and high-sensitivity cTnI (ARCHITECT STAT, Abbott Laboratories, Abbott Diagnostics) were measured on Cobas e411 and i1000SR analyzers, respectively, using the manufacturers’ calibrators and quality controls.^6^ We also participated in the UK National External Quality Assurance Scheme for these biomarkers during the conduct of study. The limit of blank of the cTnT assay is set to 3 ng/L by the manufacturer, whereas we reported anything <1.2ng/L for cTnI as below the limit of blank.^12^ Results below the limit of blank (undetectable) are reported as half of the limit of blank (ie, 1.5 ng/L for cTnT and 0.6 ng/L for cTnI) for continuous analyses. A total of 19 501 individuals provided measurements of both troponins.

### Health Outcomes

Participants were followed to the end of September 2017 for deaths or hospitalizations for events of interest. Outcomes were identified by using a national database: the Information Services Division National Health Service record linkage for Scotland. This contains information on Scotland’s morbidity records for acute specialty day case and inpatient discharges from hospital (Scotland’s morbidity record 01) since January 1981. Causes of death, derived from death certificates, were obtained from National Health Service Central. For this study, the primary composite CVD outcome was any event included in the national ASSIGN risk score definition of CVD,^13^ including any *International Classification of Diseases, 10th Revision* codes I20–25, G45, I60–69, and death from CVD (I00–I99), as well, and OPCS-4 (Office of Population Censuses and Surveys: Classification of Interventions and Procedures version 4) procedure codes L29.5, L31.1, K40–46, K49, and K75 (procedures comprising carotid endarterectomy, carotid angioplasty, coronary artery bypass graft, and percutaneous transluminal coronary angioplasty). CVD death included deaths coded from underlying causes I00 to I99; all others were classified as non-CVD deaths. Other clinical outcomes (fatal or nonfatal) included coronary heart disease (I00–I25), myocardial infarction (I21, I22), ischemic stroke (I63, I64, G45), any malignancy (C00–C97), and hospitalization for heart failure (I50, I42.0, I42.6, I42.7, I42.9, I11.0).

### Genome-Wide Association Study

Details on blood collection and DNA extraction are provided elsewhere.^14^ Samples were genotyped using the Illumina Human OmniExpressExome-8v1.0 Bead Chip and Infinum chemistry and processed using the Illumina Genome Studio Analysis software v2011 (Illumina). Quality control was performed to remove single-nucleotide polymorphisms (SNPs) with <98% call rate, individuals with a genotyping rate <98%, and SNPs with a Hardy Weinberg equilibrium test *P* value of ≤1×10^−6^ and a minor allele count of <50. Individuals who were identified as population outliers through principal component analyses of their genotypic information were also removed.^15^ Following quality control there were 19 904 GS:SFHS individuals (11 731 women and 8173 men) that had genotypic information for 561 125 autosomal SNPs, with 19 130 participants having phenotyping for at least one troponin. To increase the density of variants throughout the genome, the genotyped data were imputed using the Sanger Imputation Service (https://imputation.sanger.ac.uk/) using the Haplotype Reference Consortium panel v1.1.^16^ Autosomal haplotypes were checked to ensure consistency with the reference panel (strand orientation, reference allele, position) then prephased using Shapeit2 v2r837,^17,18^ the Shapeit2 duohmm option11,^19^ taking advantage of the cohort family structure to improve the imputation quality. Monogenic and low imputation quality (info score < 0.4) variants were removed from the imputed data set leaving 24 111 857 variants available for downstream analysis.

### Statistical Analysis for Risk Associations

The intraclass correlation coefficients for cTnT and cTnI were 0.18 (95% CI, 0.16–0.19) and 0.09 (95% CI, 0.07–0.10), respectively, for clustering within family groups. Allowing for familial clustering had no appreciable effect on any analyses, so the results presented here are from analyses without adjustment for clustering. Multiple imputation by chained equations was used to account for missing data for classical risk factors (but not missing troponin concentrations) in regression models. Ten imputed data sets were used.

To illustrate associations of troponins and classical risk factors with health outcomes, available complete data were presented using categorical variables expressed as frequencies and percentages, and continuous variables were presented as medians (interquartile interval) or mean (SD). Each troponin was split into 3 unequal groups (low: cTnT <3.0 ng/L and cTnI ≤1.8 ng/L; intermediate: cTnT 3.0–5.7 ng/L and cTnI 1.9–3.0ng/L; high: cTnT ≥5.8 ng/L and cTnI ≥3.1 ng/L), so that the proportion within each grouping was similar for each troponin. Kaplan–Meier curves were used to illustrate the association of the 3 groups of each troponin with incident CVD.

Unadjusted and adjusted (for age, sex, total cholesterol, high-density lipoprotein cholesterol, systolic blood pressure, number of cigarettes smoked per day, rheumatoid arthritis, diabetes mellitus, Scottish Index of Multiple Deprivation score, family history of CVD, cholesterol-lowering medication, blood pressure–lowering medication, and baseline CVD). Cox proportional hazards models were used to investigate the association of the troponins with health outcomes. Further adjustment for body mass index and creatinine made no substantial difference (data not shown). The shapes of the associations were tested and illustrated using restricted cubic splines (knots at 2, 3, 5, 10, and 25 pg/mL for cTnI, and at 3.3, 5, 8, 10, and 25 pg/mL for cTnT) and also modeled using troponins as continuous risk factors (per 1 SD increase after log-transformation). The proportional hazards assumption was tested by plotting Schoenfeld residuals. Tests for interaction of cTnT and cTnI with CVD events were also conducted by baseline classical risk factors. The 95% CI for the difference in the log hazard ratio for cTnI and cTnT was obtained by bootstrapping (5000 times); exponentiation of the difference in the log hazard ratios gives the ratio of the hazard ratios. Improvement in the clinical prediction of primary CVD (using age, sex, total cholesterol, high-density lipoprotein cholesterol, systolic blood pressure, number of cigarettes per day, Scottish Index of Multiple Deprivation score, diabetes mellitus, family history of CVD, and rheumatoid arthritis) on the addition of each troponin individually was tested (in participants without baseline CVD and aged ≥40 years) using the continuous net reclassification index and integrated discrimination index. These tests were conducted using the nricens package (R) with 5000 bootstraps. All analyses were performed in STATA (version 14.2) and R (version 3.3.1).

### Statistical Analysis for GWAS

GS:SFHS has previously been used in a variety of GWAS studies and the imputed data are research ready.^20,21^ There were 19 130 individuals with a troponin result and quality-controlled genomic data.

Genome-wide associations were performed on Haplotype Reference Consortium–imputed data, only results from variants with a minor allele count of 50 in our sample. For each phenotype, an additive model for the fitted SNP fixed effect was set up incorporating age and sex as covariates and a random polygenic effect accounting for the relatedness among participants. Phenotypes were inverse-normal transformed to ensure a normal distribution of the model’s residuals, using the rntransform function in the GenABEL R package.^22^ Associations with the Haplotype Reference Consortium–imputed variants were performed with the software RegScan v0.2.^23^ The pgresidualY estimated from the polygenic function in GenABEL was used for association analysis. The effect size, standard errors, and *P* values were thereafter corrected to account for relatedness using the GRAMMAR-Gamma factors also provided by the polygenic function.^24^ The polygenic command in the GenABEL R package was used to calculate Genetic kinship-based heritability. The standard errors of heritability estimates were obtained by rerunning the polygenic command and fixing the heritability to 0.

GWAS significance of SNPs is attained at a *P* value of <5×10^–8^, and suggestive hits at *P* value <1×10^–5^. Manhattan plots are used to illustrate hits using these thresholds. All suggestive hits for both troponins were read into FUMA^25^ (https://fuma.ctglab.nl) to identify loci of interest and functional annotations. The 1000-genome European reference panel (phase 3) was used to account for linkage disequilibrium in the sample. For the primary analyses, we focused only on SNPs that attained a GWAS significant association, with a further sensitivity analysis using a more relaxed criterion for exploration; the minimum value of a lead SNP was taken at a *P* value of <5×10^–7^, and without a minimum minor allele frequency, to maximize loci identification. However, interpretation of results in this sensitivity analysis should place less emphasis on rare isolated variants

## Results

### Crude Associations of Troponins With Classical Risk Factors and With CVD

Detectable concentrations of cTnI and cTnT were found in 14 579 participants (74.8%) and 10 395 participants (53.3%), respectively. Both troponins were generally associated with adverse classical CVD risk factors, with the exception that cTnI and cTnT were inversely associated with current smoking, and cTnT was inversely associated with total cholesterol (Table I in the online-only Data Supplement). The Spearman correlation coefficient between cTnT and cTnI was *r*=0.443 (*P*<0.001).

Median follow-up was 7.8 years (quartile 1 to quartile 3, 7.1–9.2) for the primary composite CVD end point. Of the 19 501 participants, 1177 experienced an incident CVD during follow-up (6.0%), 640 participants died (3.3%), and 266 died of CVD causes (1.4%). Participants who experienced the composite CVD outcome, in general, had a more adverse risk factor profile, including being older, more frequently being male, having a higher body mass index, higher blood pressure, lower high-density lipoprotein cholesterol (but also a lower total cholesterol), and a higher deprivation score; they were more frequently smokers, and were more likely to have baseline CVD, diabetes mellitus, or rheumatoid arthritis, or to be taking cholesterol- or blood pressure–lowering medication (Table 1). Those who experienced a composite CVD event had almost a 2-fold higher median baseline cTnI and cTnT (Table 1). Similar results were found for other outcomes of interest; both troponins were higher in those who experienced every adverse outcome of interest (Tables II through IX in the online-only Data Supplement). Kaplan–Meier curves show that both cTnI and cTnT were associated with the probability of CVD-free survival (both log rank tests *P*<0.001; Figure I in the online-only Data Supplement).

**Table 1.**
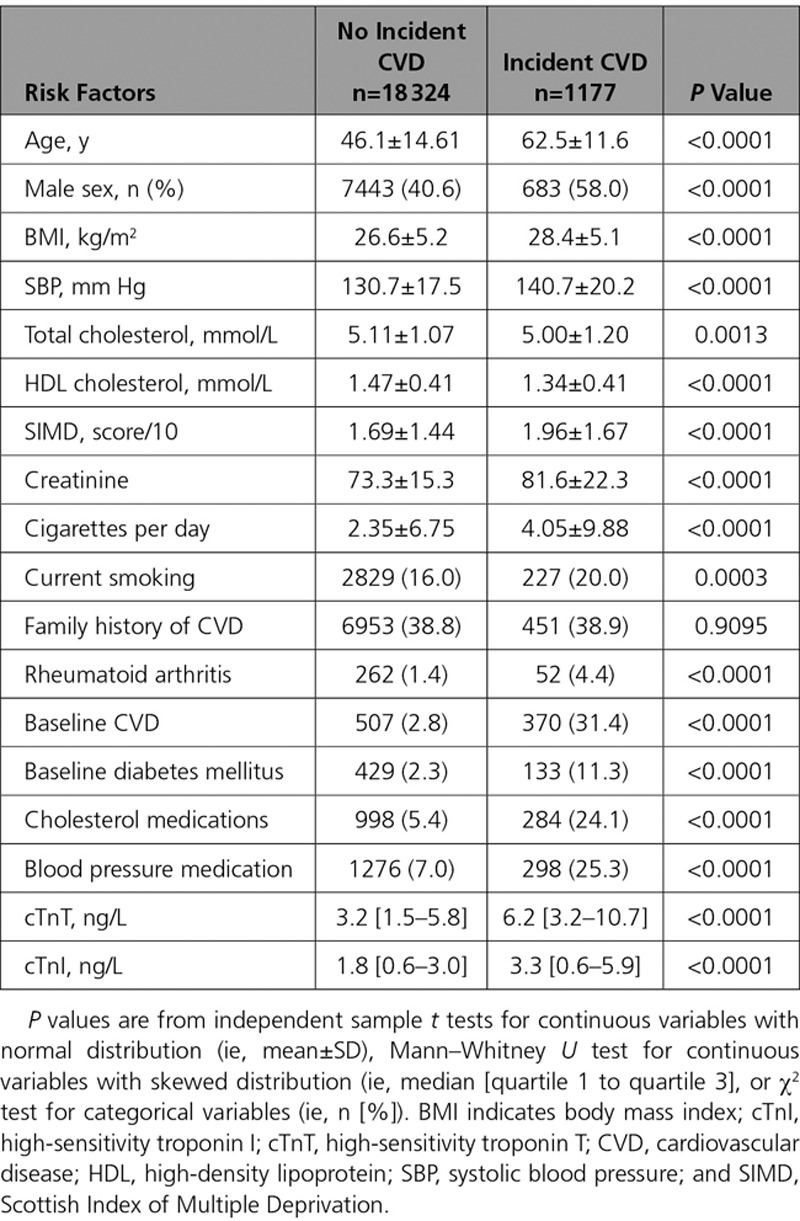
Association of Classical Risk Factors and Troponin I and Troponin T With Composite CVD Events

### Shape of the Association of Troponins With Outcomes

**Figure 1.**
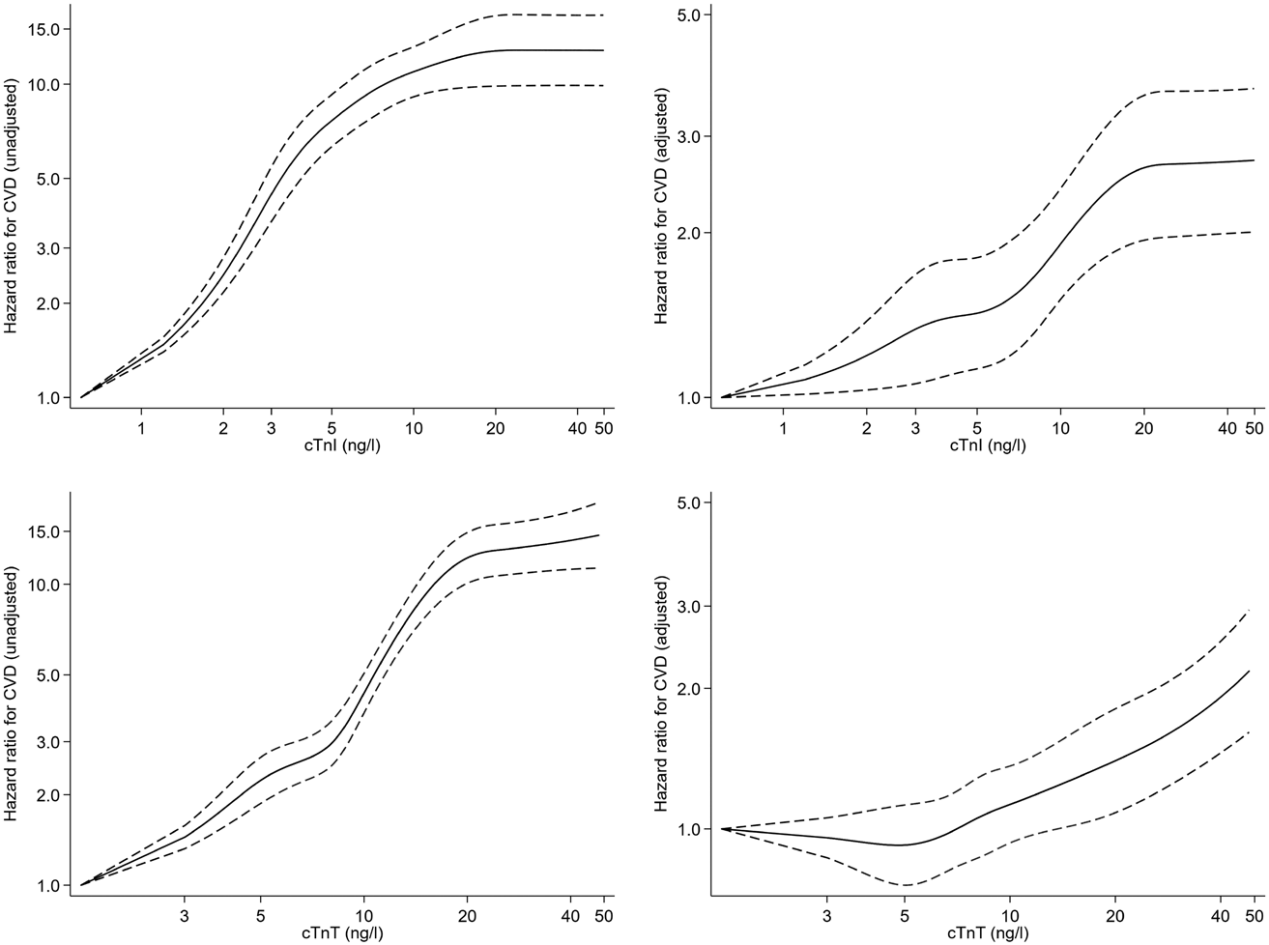
**Association of cTnI and cTnT unadjusted and adjusted (as per Table 2) with the composite CVD event.** The referent (HR=1) is undetectable levels of cTnI and cTnT, respectively. Both splines on log scale. cTnI indicates high-sensitivity cardiac troponin I; cTnT, high-sensitivity cardiac troponin T; CVD, cardiovascular disease; and HR, hazard ratio.

Using undetectable levels of each troponin as the referent, the association of elevated troponin with composite CVD was explored by restricted cubic splines (Figure 1). For both cTnI and cTnT, there was a rapid rise in risk in unadjusted models. For instance, at a cTnI level of 5 ng/L, the hazard ratio (HR) was 7.7 (95% CI, 6.3–9.3) in comparison to undetectable cTnI, and at 20ng/L, the HR was 12.8 (9.9–16.5). The corresponding HRs for cTnT were 2.2 (1.9–2.7) and 12.2 (10.1–14.9), respectively. For both troponins, there was a leveling off in increasing risk at higher levels of troponin. Adjustment for classical risk factors attenuated the associations for both troponins substantially, and made the associations more linear. In the adjusted model, at values of 5 ng/mL and 20 ng/L, the HRs were 1.5 (1.2–1.9) and 2.6 (1.9–3.4) for cTnI, and 0.97 (0.80–1.17) and 1.4 (1.1–1.8) for cTnT, respectively. The associations of both troponins with CVD death were more pronounced (Figure II in the online-only Data Supplement). Only cTnT showed trends toward a positive relationship with non-CVD death in adjusted models (Figure III in the online-only Data Supplement) and therefore had stronger associations with all-cause mortality (Figure IV in the online-only Data Supplement). Only cTnI, not cTnT, was associated with myocardial infarction (MI) and coronary heart disease (CHD; Figures V and VI in the online-only Data Supplement), and adjustment for age and sex alone was sufficient to ameliorate the association between cTnT and MI or CHD (data not shown). Both cTnI and cTnT were associated with ischemic stroke and heart failure (Figures VII and VIII in the online-only Data Supplement). After adjustment, neither troponin was associated with cancer (Figure IX in the online-only Data Supplement).

**Table 2.**
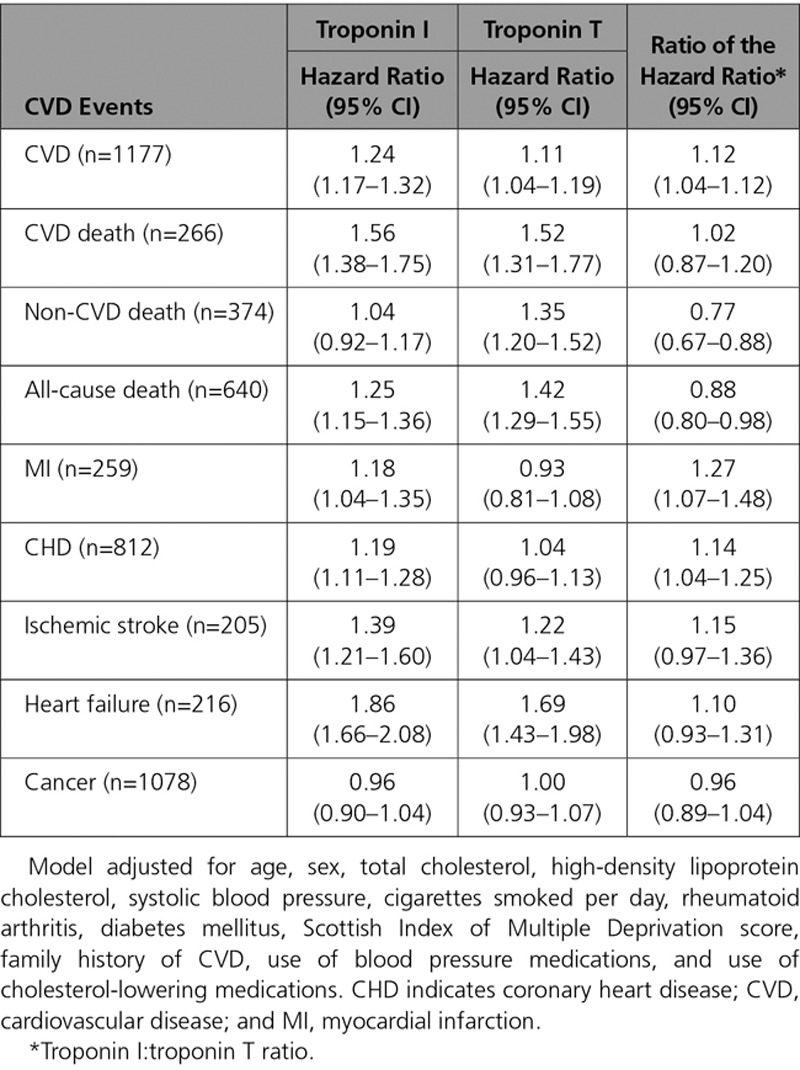
Association of Troponin I and Troponin T (per 1 SD Increase on the Log Scale) With Risk of Different Events, Adjusted for Classical Risk Factors, and the Troponins in Separate Models

**Figure 2.**
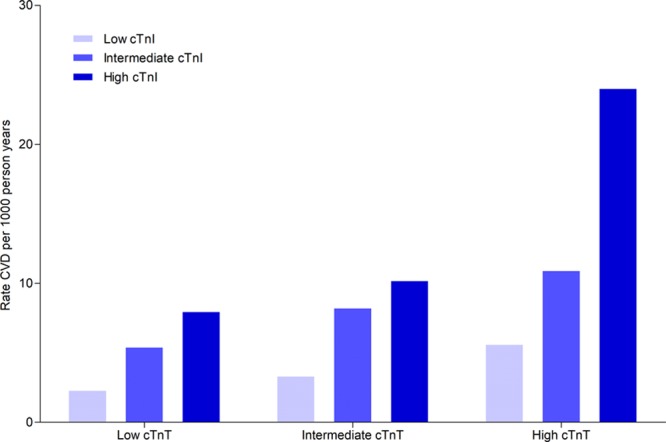
**Rates of CVD per 1000 person-years (n=1177 events) by low/intermediate/high groupings of both cTnI and cTnT.** For cTnI: low ≤1.8ng/L (n=9426), intermediate 1.9 to 3.0 ng/L (n=5052), and high ≥3.1 ng/L (n=5023). For cTnT: low ≤3.0 ng/L (n=9106), intermediate 3.0 to 5.7 ng/L (n=5200), and high ≥5.8 ng/L (n=5195). cTnI indicates high-sensitivity cardiac troponin I; cTnT, high-sensitivity cardiac troponin T; and CVD, cardiovascular disease.

A higher category (low/intermediate/high) of cTnI was associated with increased unadjusted rate of composite CVD within each category of cTnT (Figure 2). Likewise, a higher category of cTnT was associated with increased rate of composite CVD within each category of cTnI (Figure 2). After controlling for cTnT, each category increase in cTnI had a HR of 1.92 (95% CI, 1.77–2.07) for composite CVD in comparison with the preceding category. After controlling for cTnI, the HR for one category increase in cTnT was 1.64 (95% CI, 1.52–1.78).

### Comparison of the Association of cTnI and cTnT With Outcomes

We then compared the extent of the adjusted association of cTnI and cTnT with the different outcomes. One standard deviation increase in log cTnI was associated with a HR of 1.24 for composite CVD, whereas the HR was 1.11 for 1 SD increase in log cTnT. The ratio of these HRs indicates that the association with composite CVD was stronger for cTnI: 1.12 (95% CI, 1.04–1.21; Table 2). Both troponins had similarly strong associations with CVD death, and both had stronger associations with CVD death than for the composite CVD outcome (Table 2). Both troponins were also strongly associated with heart failure (Table 2). cTnT, but not cTnI, had an association with non-CVD death (ratio of HRs, 0.77; 95% CI, 0.67–0.88), and consequently cTnT also had a stronger association with all-cause death than cTnI (Table 2). In contrast, only cTnI was associated with MI or CHD; cTnT showed no association with either outcome. Both troponins showed an association with ischemic stroke, and neither was associated with incident cancer after adjustment (Table 2). When both cTnI and cTnT were included in the same adjusted model together, only cTnI remained associated with the composite CVD outcome (Table 3).

**Table 3.**
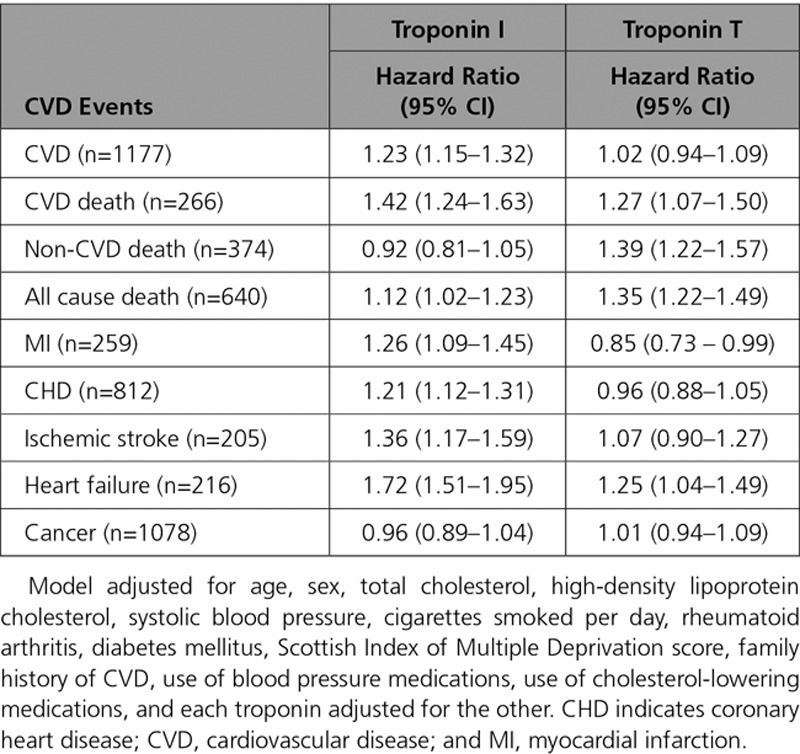
Association of Troponin I and Troponin T (per 1 SD Increase on the Log Scale) With Risk of Different Events, Adjusted, With Both Troponins in the Same Model

### Interaction by Classical Risk Factors

Investigating interactions by baseline risk factors for the composite CVD outcome, both cTnI and cTnT showed trends to be less strongly associated with composite CVD among those with baseline CVD or taking blood pressure– or cholesterol-lowering medications (Figure X in the online-only Data Supplement). cTnI was more strongly associated with composite CVD risk among patients with diabetes mellitus (*P* for interaction 0.009; Figure X in the online-only Data Supplement). There were no other notable interactions.

### Primary Composite CVD Prediction Models

Using the continuous net reclassification index, a clinical prediction model in those without baseline CVD and aged ≥40 years (n=12 496) was improved by the addition of cTnI which yielded a continuous net reclassification index improvement of 7.7% (95% CI, 2.8%–11.7%; *P*=0.004), and an integrated discrimination index of +0.005 (95% CI, 0.002–0.009; *P*<0.001). The clinical prediction model also showed a trend to be improved by addition of cTnT separately, yielding a continuous net reclassification index improvement of 8.0% (95% CI, –2.3% to 11.9%; *P*=0.064), and an integrated discrimination index of +0.001 (95% CI, 0.000–0.002; *P*=0.001).

### GWAS for cTnI and cTnT

**Figure 3.**
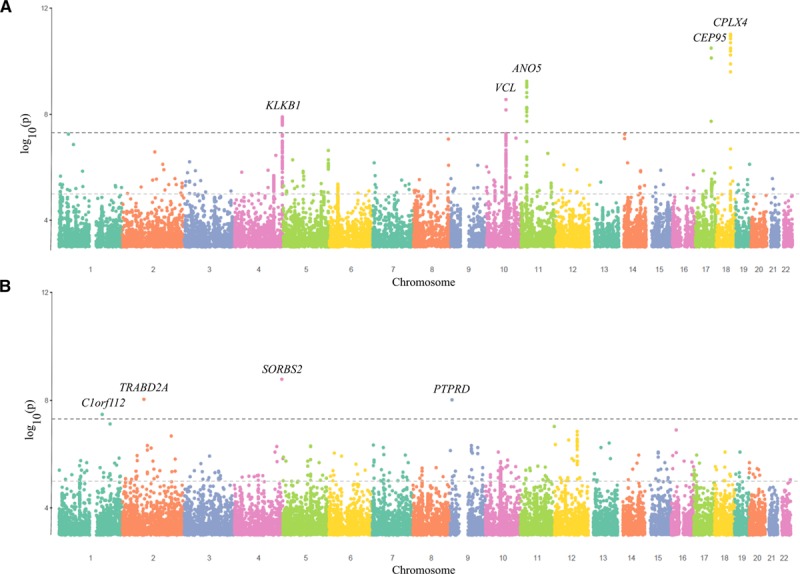
**Manhattan plots for single-nucleotide polymorphisms associated with cTnI and cTnT after adjustment for age and sex (n=19 130).** **A**, cTnI. **B**, cTnT. The horizontal black dotted line indicates genome-wide significance at *P*<5×10–8, and the horizontal gray dotted line suggests significance at *P*<1×10–5.

Heritability of cTnI was 0.249±0.013 and cTnT was 0.35±0.011. There were 5 loci for cTnI that reached genome-wide significance (Figure 3A). These included SNPs at *KLKB1* (4q35.2: 23 SNPs at GWAS significance), *VCL/AP3M1/ADK* (10q22.2: 2 SNPs), *ANO5* (11p14.3: 14 SNPs), *CEP95/SMURF2* (17q23.3: 3 SNPs), and *LMAN1/CPLX4* (18q21.32: 11 SNPs) genes (Table 4). There was an isolated intergenic SNP in chromosome 1 that was of borderline genome-wide significance, flanking a suggestive hit for *DAB1* (1p32.1) SNP. There were marked differences in the GWAS Manhattan plot for cTnT in comparison to cTnI (Figure 3B), with limited overlap of associated loci, and far fewer suggestive loci associated with cTnT in general. There were isolated low-frequency SNPs at the genes of *C1orf112*, *TRABD2A* (2p11.2), *SORBS2* (4q35.1), and *PTPRD* (9p23) for cTnT (Table 4).

**Table 4.**
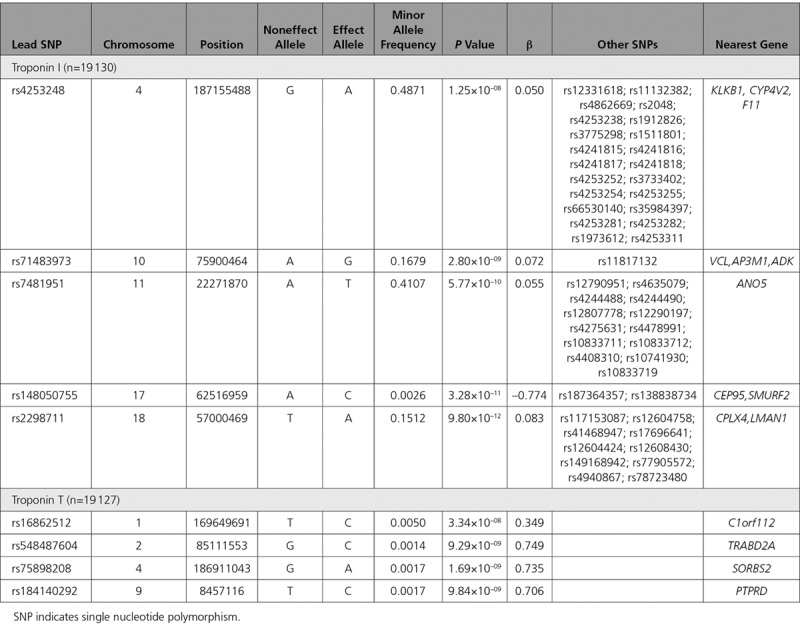
Lead SNPs, and Other SNPs in the Same Loci, With Genome-Wide Association Study Significant Associations With Either Troponin (at *P*<5×10^–8^)

Using a more relaxed threshold for statistical significance identified other loci of potential interest. For cTnI, a locus at *F12/GRK6* (5q35.3: 5 SNPs) also showed suggestive trends toward association (Table X in the online-only Data Supplement). For cTnT, there was also a suggestive hit for a locus at *POC1B* (12q21.33: 16 SNPs) and *TMEM131/*ZAP70 (2q11.1: 4 SNPs). Excluding those with baseline CVD had minimal impact on the primary genetic associations (Table XI in the online-only Data Supplement).

## Discussion

This study highlights several important findings that are relevant to potential future clinical use of high-sensitivity troponins in CVD risk prediction, and also relevant to scientific enquiries into the etiology of elevated troponin in apparently healthy people. It is most remarkable that we demonstrate the striking differences in cTnI and cTnT in terms of their association with composite CVD and with specific CVD outcomes. Both have similar strong associations with risk of CVD death and heart failure, and both have associations with ischemic stroke. It is most surprising, however, that cTnT showed no association with MI or CHD after adjusting for classical risk factors; cTnI did. As a result, cTnI was more strongly associated with the primary composite CVD outcome. In fact, cTnT showed an association with non-CVD death but cTnI did not. These findings suggest different upstream causes of modest elevations in cTnI and cTnT in the general population. In line with this, our GWAS highlights little overlap in the genetic determinants of circulating cTnI and cTnT. This is in line with our previous report, demonstrating differences in the associations of classical risk factors with the troponins in a cross-sectional study.^6^

In a recent study level meta-analysis,^4^ in the top versus bottom third of the population for each troponin, the HR of CVD was nominally stronger for cTnT than cTnI (HR=1.60 versus 1.36; *P*=0.171), and cTnT was more strongly associated with fatal CVD (*P*=0.027). However, the troponins were measured in different cohorts, which limits the ability to make direct comparisons. Our study is the first large general population study of which we are aware to compare systematically the disease associations of the 2 troponins directly.

The lack of association of cTnT with MI and CHD, after adjusting for classical risk factors, is unexpected, given that the source of both troponins is cardiac injury. Indeed, previous studies demonstrate that cTnT is associated with left ventricular hypertrophy^26^ and coronary plaque count, and plaque phenotype, as well.^27,28^ The MI and CHD outcomes were associated with cTnT in unadjusted models, and with cTnI in adjusted models, so the lack of association is not explained by outcome misspecification. Given the additional association of cTnT with fatal non-CVD events, it is interesting to speculate what causes cTnT elevation beyond classical risk factors in apparently healthy people. cTnT is known to be transiently expressed in fetal skeletal muscle,^29^ and it may be possible that noncardiac tissues express cTnT in some circumstances. For instance, patients with neuromuscular diseases but with no evidence of heart disease may have elevated cTnT, but not an elevated cTnI.^30–32^ cTnT is also known to be a strong predictor of adverse noncardiac outcome following abdominal surgery.^33^ As such, different etiological causes of elevations in cTnI and cTnT, and consequently different downstream risks, may be sufficient to explain our findings.

Upstream genetic determinants of the troponins also appear distinct. For cTnI, the *KLKB1* and *F12* genes are both part of the kallikrein-kinin axis and loci have been associated with biomarkers of endothelin-1, proadrenomedullin,^34^ B-type natriuretic peptide,^35^ and l-arginine, as well.^36^ Given this broad association with vasoactive peptides, it is interesting that the loci are associated with cTnI, but no association is seen for cTnT. Plasma prokallikrein is activated by active factor XII (encoded by *F12*) and colocalizes to endothelial cells. Activated kallikrein is also involved in procession vasoactive peptides such as bradykinin and renin.^37^ The loci around VCL encode vinculin membrane–associated protein, which is perhaps particularly relevant to troponin expression, because it appears involved in cytoskeletal modeling in diseased cardiac tissue.^38,39^ The anoctamin-5 protein encoded by *ANO5 i*s a poorly characterized chloride channel found in skeletal and cardiac muscle, and mutations may be associated with cardiomyopathy.^40^ Allelic variants of *LMAN1* gene are associated with factor V-factor VIII deficiency.^41^ In contrast, genes associated with cTnT include *ArgBP2* (*SORBS2*), which is highly abundant in cardiac z-disc structures.^42^ There is hence a strong biological background for many of these genes being associated with cardiac injury or troponin leakage.

Strengths of the study include the use of a large and well-phenotyped prospective nationwide population study of broadly healthy people, where national record linkage has been used for follow-up. Numbers of outcomes lend the study considerable statistical power. The direct comparisons of cTnI and cTnT in terms of risk associations in a general population is novel, and is supported by the GWAS in a cohort with an established approach to such studies. Weaknesses include the family structure of the study, although sensitivity analyses suggest family clustering had very limited impact on data in terms of clustering within families. The study population is not ethnically diverse, limiting generalizability in that regard, but includes a wide age range from participants of both sexes. In common with many electronic health records, the small proportion of the population who emigrate from Scotland^43^ will not have recorded outcomes of interest, but might still be considered at risk in our models. This may slightly bias estimates, but is unlikely to affect the direct comparison between biomarkers. A large proportion of participants had undetectable troponin. This is suboptimal for continuous statistical analyses, but we demonstrate results using a number of different models. We also acknowledge that, in individual patients, as opposed to population studies, accurate measurement of low troponin levels would be important in using troponin levels to determine clinical risk. Our observations offer limited causal insight as to the reasons underlying differential associations of cTnI and cTnT with different outcomes. However, etiological differences in the upstream causes of different troponin elevations are supported by GWAS, and, as such, our data are sufficient to indicate that care should be taken when selecting a high-sensitivity troponin to use as a biomarker in general population studies, or as surrogate biomarkers in trials, depending on the aims of the study.

In conclusion, we demonstrate that routine clinical cTnI and cTnT assays have different associations with composite cardiovascular and non-CVD mortality outcomes in apparently healthy people; the cTnI assay is specific for CVD risk, whereas the cTnT assay is more strongly associated with non-CVD mortality. It also appears that their genetic determinants are largely distinct. Future research studies should use this evidence base to select a troponin for cohort phenotyping depending on the study aims. This information is also of relevance to attempts to improve CVD risk stratification in the population by using cardiac troponin measurements.

## Acknowledgments

We thank J. Cooney and P. Stewart (University of Glasgow, UK) for excellent technical support. We are grateful to all the families who took part, the general practitioners, and the Scottish School of Primary Care for their help in recruiting them, and the whole Generation Scotland team, which includes interviewers, computer and laboratory technicians, clerical workers, research scientists, volunteers, managers, receptionists, healthcare assistants, and nurses.

## Sources of Funding

Troponin measurements and analysis were supported by a Stratified Medicine Grant from the Chief Scientist Office of the Scottish Government Health Directorates (ASM/14/1). Generation Scotland received core support from the Chief Scientist Office of the Scottish Government Health Directorates (CZD/16/6) and the Scottish Funding Council (HR03006). Genotyping of the samples was performed by the Genetics Core Laboratory at the Clinical Research Facility, University of Edinburgh, Edinburgh, Scotland, and was funded by the Medical Research Council UK. Dr Preiss is supported by a University of Oxford British Heart Foundation Center of Research Excellence Senior Transition Fellowship (RE/13/1/30181). Dr Mills is supported by a Butler British Heart Foundation Senior Clinical Research Fellowship (FS/16/14/32023). Dr Hayward is supported by a Medical Research Council University Unit Program Grant “QTL in Health and Disease” MC_PC_U127592696.

## Disclosures

Dr Welsh has received grant support from Roche and Boehringer Ingelheim. Dr Shah has received honoraria from Abbott Diagnostics. Dr Mills has acted as a consultant for Abbott Diagnostics, Roche, Beckman-Coulter, and Singulex. Dr Sattar has received fees for consulting, speaking, and honoraria from Roche Diagnostics, Astrazeneca, Boehringer Ingelheim, NovoNordisk, Amgen, Sanofi/Regeneron, and Janssen. The other authors report no conflicts.

## Supplementary Material
