# Supplementary material for: Cardiac Troponin T and Troponin I in the General Population: Comparing and Contrasting Their Genetic Determinants and Associations With Outcomes
Source: Circulation. 2019 Apr 24;139(24):2754–64. doi: 10.1161/CIRCULATIONAHA.118.038529 (PMC6571179; doi:10.1161/CIRCULATIONAHA.118.038529)

SUPPLEMENTAL MATERIAL

Welsh et al

Cardiac Troponin T and Troponin I in the general population: comparing and contrasting genetic determinants and their associations with outcomes

Supplemental Table 1: Classical CVD risk factors by categories of cTnI and cTnT (low; cTnT ≤3ng/L& cTnI ≤1.8ng/L, intermediate; cTnT 3.01-5.79ng/L & cTnI 1.9-3.0ng/L, high cTnT ≥5.8ng/L & cTnI ≥3.1ng/L)

	cTnI				cTnT			
	Low cTnI	Intermediate cTnI	High cTnI	p-value	Low cTnT	Intermediate cTnT	High cTnT	p-value
	n=9426	n=5052	N=5023		n=9106	n=5200	n=5195	
Age	41.2 (13.5)	50.4 (13.3)	54.7 (14.6)	<0.0001	42.2 (13.0)	48.2 (14.2)	54.5 (15.7)	<0.0001
Male sex	2628 (27.9%)	2401 (47.5%)	3097 (61.7%)	<0.0001	2557 (28.1%)	2507 (48.2%)	3062 (58.9%)	<0.0001
Body mass index (kg/M2)	25.8 (5.1)	27.2 (5.0)	27.8 (5.2)	<0.0001	26.2 (5.2)	26.9 (5.1)	27.3 (5.2)	<0.0001
Systolic blood pressure (mmHg)	125.1 (15.2)	134.7 (16.9)	139.6 (18.8)	<0.0001	126.9 (16.1)	133.1 (17.4)	137.4 (18.9)	<0.0001
Total cholesterol (mmol/L)	4.96 (1.03)	5.30 (1.10)	5.15 (1.11)	<0.0001	5.10 (1.06)	5.16 (1.10)	5.04 (1.10)	<0.0001
HDL cholesterol (mmol/L)	1.49 (0.40)	1.45 (0.42)	1.42 (0.41)	<0.0001	1.48 (0.40)	1.45 (0.41)	1.44 (0.42)	<0.0001
SIMD score	12 (7, 24)	11 (7, 22)	11 (7, 22)	<0.0001	12 (7, 24)	11 (7, 21)	11 (7, 20)	<0.0001
Creatinine (μmol/L)	70.3 (12.7)	74.2 (14.8)	79.9 (20.0)	<0.0001	71.0 (12.4)	73.9 (14.1)	78.7 (21.0)	<0.0001
Current smoker	1759 (19.3%)	735 (15.1%)	562 (11.5%)	<0.0001	1719 (19.5%)	705 (14.1%)	632 (12.5%)	<0.0001
Family history of heart disease	3629 (39.3%)	1912 (38.8%)	1863 (37.8%)	0.184	3497 (39.2%)	2000 (39.4%)	1907 (37.4%)	0.0476

Rheumatoid arthritis	112 (1.2%)	92 (1.8%)	110 (2.2%)	<0.0001	113 (1.2%)	88 (1.7%)	113 (2.2%)	<0.0001
Baseline heart disease or stroke	184 (2.0%)	209 (4.1%)	484 (9.6%)	<0.0001	184 (2.0%)	217 (4.2%)	476 (9.2%)	<0.0001
Baseline diabetes	188 (2.0%)	132 (2.6%)	242 (4.8%)	<0.0001	118 (1.3%)	143 (2.8%)	301 (5.8%)	<0.0001
Baseline use of cholesterol lowering medications	224 (2.4%)	401 (7.9%)	657 (13.1%)	<0.0001	260 (2.9%)	344 (6.6%)	678 (13.1%)	<0.0001
Baseline use of blood pressure lowering medications	263 (2.8%)	471 (9.3%)	840 (16.7%)	<0.0001	330 (3.6%)	445 (8.6%)	799 (15.4%)	<0.0001

Values are mean (sd) for continuous variables (median and Q1-Q3 for SIMD), and n (%) for categorical variables
P-values from ANOVA for continuous variables (excepting SIMD, which is by Kruskal-Wallis test) and chi-squared for categorical variables

Supplemental Table 2: Association of classical risk factors and Troponin I and Troponin T with death from CVD causes

	**Did not die from CVD causes N=19235**	**Died from CVD causes N=266**	**p-value**
Age (years)	46.8±14.8	69.1±12.2	<0.0001
Male sex	7988(41.5%)	138(51.9%)	0.0007
BMI (kg/m2)	26.6±5.2	28.0±5.4	<0.0001
SBP (mmHg)	131.2±17.7	143.7±21.7	<0.0001
Total cholesterol (mmol/L)	5.11±1.08	4.69±1.16	<0.0001
HDL cholesterol (mmol/L)	1.46±0.41	1.36±0.43	<0.0001
SIMD (score/10)	1.70±1.45	2.06±1.68	<0.0001
Creatinine	73.6±15.5	88.2±30.2	<0.0001
Cigarettes per day	2.4±6.9	4.9±12.8	<0.0001
Current smoking	3004(16.1%)	52(20.5%)	0.0630
Family History of CVD	7312(38.8%)	92(35.0%)	0.2019
Rheumatoid arthritis	300(1.6%)	14(5.3%)	<0.0001
Baseline CVD	785(4.1%)	92(34.6%)	<0.0001
Baseline diabetes	519(2.7%)	43(16.2%)	<0.0001
Cholesterol medications	1215(6.3%)	67(25.2%)	<0.0001
Blood pressure medication	1494(0.078)	80(0.301)	<0.0001
hsTnT (ng/L)	3.3[1.5,5.9]	9.3 [4.9,16.3]	<0.0001
hsTnI (ng/L)	1.9[0.6,3.1]	4.9 [2.7,8.9]	<0.0001

Supplemental Table 3: Association of classical risk factors and Troponin I and Troponin T with non-CVD mortality

	Did not die, or died of CVD N=19127	Died from non-CVD causes N=374	p-value
Age (years)	46.7±14.8	63.1±12.4	<0.0001
Male sex	7958(41.6%)	168(44.9%)	0.198
BMI (kg/m2)	26.6±5.2	27.4±5.4	0.0069
SBP (mmHg)	131.2±17.7	141.6±22.0	<0.0001
Total cholesterol (mmol/L)	5.10±1.08	5.12±1.15	0.7729
HDL chol (mmol/L)	1.46±0.41	1.48±0.47	0.285
SIMD (score/10)	1.70±1.45	1.93±1.64	0.0024
Creatinine	73.7±15.7	79.7±25.3	<0.0001
Cigarettes per day	2.4±6.9	5.4±11.8	<0.0001
Current smoking	2968(16.0%)	88(24.6%)	<0.0001
Family History of CVD	7274(38.9%)	130(35.1%)	0.1453
Rheumatoid arthritis	294(1.5%)	20(5.3%)	<0.0001
Baseline CVD	838(4.4%)	39(10.4%)	<0.0001
Baseline diabetes	528(2.8%)	34(9.1%)	<0.0001
Cholesterol medications	1238(6.5%)	44(11.8%)	<0.0001
Blood pressure medication	1525(8.0%)	49(13.1%)	0.0003
hsTnT (ng/L)	3.3[1.5,5.9]	6.4 [3.8,10.5]	<0.0001
hsTnI (ng/L)	1.9[0.6,3.1]	3.0 [1.8,4.9]	<0.0001

Supplemental Table 4: Association of classical risk factors and Troponin I and Troponin T with all cause mortality

	**Did not die N=18861**	**Died N=640**	**p-value**
Age (years)	46.4±14.6	65.6±12.7	<0.0001
Male sex	7820(41.5%)	306(47.8%)	0.0013
BMI (kg/m2)	26.6±5.2	27.6±5.4	<0.0001
SBP (mmHg)	131.0±17.5	141.9±21.9	<0.0001
Total cholesterol (mmol/L)	5.11±1.08	4.94±1.17	0.0001
HDL chol (mmol/L)	1.46±0.41	1.43±0.46	0.0727
SIMD (score/10)	1.69±1.45	1.99±1.66	<0.0001
Creatinine	73.5±15.3	83.2±27.7	<0.0001
Cigarettes per day	2.4±6.7	5.2±12.2	<0.0001
Current smoking	2916(16.0%)	140(22.9%)	<0.0001
Family History of CVD	7182(38.9%)	222(35.1%)	0.0509
Rheumatoid arthritis	280(1.5%)	34(5.3%)	<0.0001
Baseline CVD	746(4.0%)	131(20.5%)	<0.0001
Baseline diabetes	485(2.6%)	77(12.0%)	<0.0001
Cholesterol medications	1171(6.2%)	111(17.3%)	<0.0001
Blood pressure medication	1445(7.7%)	129(20.2%)	<0.0001
hsTnT (ng/L)	3.2[1.5,5.8]	7.5 [4.1,12.3]	<0.0001
hsTnI (ng/L)	1.9[0.6,3.0]	3.6 [2.0,6.2]	<0.0001

Supplemental Table 5: Association of classical risk factors and Troponin I and Troponin T with myocardial infarction

	**No incident MI N=19242**	**Incident MI N=259**	**p-value**
Age (years)	46.9±14.9	60.8±10.5	<0.0001
Male sex	7948(41.3%)	178(68.7%)	<0.0001
BMI (kg/m2)	26.6±5.2	28.7±5.3	<0.0001
SBP (mmHg)	131.2±17.8	141.1±18.3	<0.0001
Total cholesterol (mmol/L)	5.10±1.08	5.39±1.24	<0.0001
HDL cholesterol (mmol/L)	1.46±0.41	1.27±0.36	<0.0001
SIMD (score/10)	1.69±1.45	2.13±1.69	<0.0001
Creatinine	73.7±15.8	80.8±19.6	<0.0001
Cigarettes per day	2.4±7.0	4.2±9.1	<0.0001
Current smoking	3001(16.1%)	55(22.0%)	0.0123
Family History of CVD	7305(38.8%)	99(38.5%)	0.9294
Rheumatoid arthritis	307(1.6%)	7(2.7%)	0.1596
Baseline CVD	821(4.3%)	56(21.6%)	<0.0001
Baseline diabetes	532(2.8%)	30(11.6%)	<0.0001
Cholesterol medications	1232(6.4%)	50(19.3%)	<0.0001
Blood pressure medication	1526(7.9%)	48(18.5%)	<0.0001
hsTnT (ng/L)	3.3[1.5,6.0]	5.3 [1.5,10.0]	<0.0001
hsTnI (ng/L)	1.9[0.6,3.1]	3.1 [2.0,5.2]	<0.0001

Supplemental Table 6: Association of classical risk factors and Troponin I and Troponin T with coronary heart disease

	**No incident CHD n=18689**	**Incident CHD N=812**	**p-value**
Age (years)	46.4±14.8	62.1±10.7	<0.0001
Male sex	7622(40.8%)	504(62.1%)	<0.0001
BMI (kg/m2)	26.6±5.2	28.7±5.1	<0.0001
SBP (mmHg)	130.9±17.6	140.6±20.0	<0.0001
Total cholesterol (mmol/L)	5.10±1.07	4.99±1.21	0.0024
HDL cholesterol (mmol/L)	1.47±0.41	1.30±0.39	<0.0001
SIMD (score/10)	1.69±1.44	1.97±1.69	<0.0001
Creatinine	73.5±15.5	81.7±22.8	<0.0001
Cigarettes per day	2.4±6.9	3.69±9.0	<0.0001
Current smoking	2907(16.1%)	149(19.1%)	0.0271
Family History of CVD	7093(38.8%)	311(38.9%)	0.9363
Rheumatoid arthritis	283(1.5%)	31(3.8%)	<0.0001
Baseline CVD	566(3.0%)	311(38.3%)	<0.0001
Baseline diabetes	463(2.5%)	99(12.2%)	<0.0001
Cholesterol medications	1070(5.7%)	212(26.1%)	<0.0001
Blood pressure medication	1370(7.3%)	204(25.1%)	<0.0001
hsTnT (ng/L)	3.2[1.5,5.9]	6.1 [3.3,10.4]	<0.0001
hsTnI (ng/L)	1.9[0.6,3.0]	3.4 [2.1,5.6]	<0.0001

Supplemental Table 7: Association of classical risk factors and Troponin I and Troponin T with ischaemic stroke

	**No incident ischaemic stroke N=19296**	**Incident ischaemic stroke N=205**	**p-value**
Age (years)	46.9±14.9	61.7±13.1	<0.0001
Male sex	8014(41.5%)	112(54.6%)	0.0002
BMI (kg/m2)	26.7±5.2	27.7±5.0	0.0059
SBP (mmHg)	131.2±17.7	143.6±21.9	<0.0001
Total cholesterol (mmol/L)	5.10±1.08	5.11±1.22	0.9122
HDL cholesterol (mmol/L)	1.46±0.41	1.40±0.46	0.0453
SIMD (score/10)	1.70±1.45	1.90±1.60	0.0617
Creatinine	73.7±15.8	81.9±19.9	<0.0001
Cigarettes per day	2.4±7.0	4.6±9.3	<0.0001
Current smoking	3008(16.1%)	48(24.1%)	0.0023
Family History of CVD	7327(38.8%)	77(38.5%)	0.9329
Rheumatoid arthritis	303(1.6%)	11(5.4%)	<0.0001
Baseline CVD	838(4.3%)	39(19.0%)	<0.0001
Baseline diabetes	539(2.8%)	23(11.2%)	<0.0001
Cholesterol medications	1243(6.4%)	39(19.0%)	<0.0001
Blood pressure medication	1522(7.9%)	52(25.4%)	<0.0001
hsTnT (ng/L)	3.3[1.5,6.0]	6.4 [3.1,11.5]	<0.0001
hsTnI (ng/L)	1.9[0.6,3.1]	3.6 [2.1,7.0]	<0.0001

Supplemental Table 8: Association of classical risk factors and Troponin I and Troponin T with heart failure (HF)

	**No HF N=19285**	**HF N=216**	**p-value**
Age (years)	46.9±14.9	64.7±13.1	<0.0001
Male sex	7994(41.5%)	132(61.1%)	<0.0001
BMI (kg/m2)	26.6±5.2	28.0±6.1	<0.0001
SBP (mmHg)	131.2±17.7	141.4±21.5	<0.0001
Total cholesterol (mmol/L)	5.10±1.08	4.82±1.19	0.0001
HDL cholesterol (mmol/L)	1.46±0.41	1.38±0.46	0.0023
SIMD (score/10)	1.70±1.46	1.73±1.46	0.8024
Creatinine	73.6±15.6	88.1±30.2	<0.0001
Cigarettes per day	2.4±6.9	4.0±10.0	0.0018
Current smoking	3018(16.2%)	38(18.4%)	0.3976
Family History of CVD	7324(38.8%)	80(37.6%)	0.7110
Rheumatoid arthritis	303(1.6%)	11(5.1%)	<0.0001
Baseline CVD	791(4.1%)	86(39.8%)	<0.0001
Baseline diabetes	530(2.7%)	32(14.8%)	<0.0001
Cholesterol medications	1236(6.4%)	46(21.3%)	<0.0001
Blood pressure medication	1530(7.9%)	44(20.4%)	<0.0001
hsTnT (ng/L)	3.3[1.5,6.0]	9.1 [4.4,14.3]	<0.0001
hsTnI (ng/L)	1.9[0.6,3.1]	5.1 [2.9,10.2]	<0.0001

Supplemental Table 9: Association of classical risk factors and Troponin I and Troponin T with cancer

	**No cancer N=18423**	**Cancer N=1078**	**p-value**
Age (years)	46.3±14.8	59.5±11.4	<0.0001
Male sex	7675(41.7%)	451(41.8%)	0.9089
BMI (kg/m2)	26.6±5.2	27.5±5.2	<0.0001
SBP (mmHg)	131.0±17.6	137.8±19.7	<0.0001
Total cholesterol (mmol/L)	5.09±1.08	5.22±1.12	0.0001
HDL cholesterol (mmol/L)	1.46±0.41	1.48±0.45	0.0613
SIMD (score/10)	1.70±1.45	1.72±1.49	0.7208
Creatinine	73.7±15.8	76.1±18.3	<0.0001
Cigarettes per day	2.4±6.8	3.4±9.0	<0.0001
Current smoking	2875(16.1%)	181(17.5%)	0.2425
Family History of CVD	6986(38.8%)	418(39.4%)	0.6758
Rheumatoid arthritis	287(1.6%)	27(2.5%)	0.0164
Baseline CVD	767(4.2%)	110(10.2%)	<0.0001
Baseline diabetes	508(2.8%)	54(5.0%)	<0.0001
Cholesterol medications	1144(6.2%)	138(12.8%)	<0.0001
Blood pressure medication	1411(7.7%)	163(15.1%)	<0.0001
hsTnT (ng/L)	3.2[1.5,5.9]	4.7 [1.5,8.1]	<0.0001
hsTnI (ng/L)	1.9[0.6,3.0]	2.5 [1.5,4.0]	<0.0001

Supplemental Table 10. Sensitivity analysis for GWAS: lead SNPs with a p-value ≤$5x10^{-7}$ (GWAS significance attained at $5x10^{-8}$) and other SNPs in the same loci (at p-value ≤$1x10^{-5}$) associated with both troponins

**Lead SNP**	**Chromosome**	**Position**	**Non-effect allele**	**Effect allele**	**Minor allele frequency**	**P-value**	**Beta**	**Other SNPs**	**Nearest gene**
**Cardiac troponin I (n=19130)**									
rs542754544	1	37792490	G	T	0.0028	$5.65X10^{-08}$	-0.519		*Intergenic*
rs542241509	1	58174664	T	G	0.0014	$1.39X10^{-07}$	0.756		*DAB1*
rs140692556	2	128218873	C	T	0.0013	$2.70X10^{-07}$	0.710		*IWS1*
rs187147685	4	161910456	G	T	0.0015	$3.58X10^{-07}$	0.652		*intergenic*
rs4253248	4	187155488	G	A	0.4871	$1.25X10^{-08}$	0.0497	rs7682918; rs7683424; rs7661323; rs7683132; rs7684025; rs12507156; rs4862662; rs4862663; rs62350519; rs2292424; rs7687818; rs3817184; rs28698123; rs6835646; rs6810892; rs10000459; rs13106826; rs7667777; rs7377304; rs2276918; rs1053094; rs12331051; rs12331618; rs28797242; rs11132382; rs4862669; rs2048; rs4253238; rs1912826; rs3775298; rs1511801; rs1511802; rs4241815; rs4241816; rs4241817; rs4241818; rs4253252; rs3733402; rs4253253; rs4253254; rs4253255; rs66530140; rs35984397; rs4253281; rs4253282; rs80177406; rs2175495; rs1973612; rs4253295; rs4253297; rs2304595; rs4253303; rs4253304; rs4253305; rs4253308; rs4253311; rs2292423; rs4253320; rs3775303; rs4241822; rs12186258	*KLKB1, CYP4V2, F11*
rs17876031	5	176831119	A	G	0.3568	$2.29X10^{-07}$	0.048	rs1801020; rs4976691; rs2731674; rs2731673; rs2545801; rs2731672	*F12, GRK6*
rs143593851	8	137224801	G	A	0.0014	$8.54X10^{-08}$	0.773		*intergenic*
rs71483973	10	75900464	A	G	0.1679	$2.80X10^{-09}$	0.072	rs3750862; rs10824069; rs4746172; rs17743339; rs7912297; rs2270552; rs2131959; rs767809; rs2131957; rs1874151 rs1874150; rs3793921; rs2270551; rs16931177; rs2131956; rs34885185; rs10824071; rs10824072; rs10824073; rs6480716; rs7922894; rs12572820; rs11597732 rs703258; rs6480717; rs6480718; rs3180427; rs6688; rs7080130; rs7067855; rs7073435; rs7099026; rs11000882; rs7919821; rs10762576; rs2395086; rs6480719;	*VCL,AP3M1,ADK*

								rs11817132; rs4638254; rs6480721; rs10762577; rs7082003; rs7912162; rs7894143; rs67594352; rs10824095; rs10733895; rs4745741; rs10762581; rs7915999	
rs7080536	10	115348046	G	A	0.0483	$8.05X10^{-08}$	-0.110		*HABP2*
rs7481951	11	22271870	A	T	0.4107	$5.77X10^{-10}$	0.055	rs10741930; rs10833719; rs12574283; rs4338529; rs4500488; rs10766930; rs10833732; rs10833733; rs10766931; rs10741935; rs10833736; rs10766935	*ANO5*
rs499423	11	104727553	T	A	0.4422	$2.98X10^{-07}$	0.057		*Intergenic*
rs61971809	14	22057505	T	C	0.0236	$5.63X10^{-08}$	-0.160	rs61971816	*Intergenic*
rs148050755	17	62516959	A	C	0.0026	$3.28X10^{-11}$	-0.774	rs187364357; rs138838734	*CEP95,SMURF2*
rs2298711	18	57000469	T	A	0.1512	$9.80X10^{-12}$	0.083	rs117153087; rs12604758; rs41523748; rs41468947; rs17696641; rs12604424; rs12608430; rs149168942; rs77905572; rs4940867; rs78723480	*CPLX4,LMAN1*
**Cardiac Troponin T**									
rs16862512	1	169649691	T	C	0.0050	$3.34X10^{-08}$	0.349		*C1orf112*
rs184837031	1	201080174	G	A	0.0025	$7.75X10^{-08}$	-0.531	rs80092188	*CACNA1S*
rs548487604	2	85111553	G	C	0.0014	$9.29X10^{-09}$	0.749		*TRABD2A*
rs147501248	2	98598992	T	C	0.0073	$4.73X10^{-07}$	0.277	rs150582665	*TMEM131, ZAP70*
rs75898208	4	186911043	G	A	0.0017	$1.69X10^{-09}$	0.735		*SORBS2*
rs117749317	7	4274444	G	A	0.0287	$4.70X10^{-07}$	0.135	rs75274073	*SDK1*
rs184140292	9	8457116	T	C	0.0017	$9.84X10^{-09}$	0.706		*PTPRD*
rs11223971	11	134600907	G	C	0.2981	$9.24X10^{-08}$	0.0515		*intergenic*
rs568331886	12	3570021	G	A	0.0018	$4.34X10^{-07}$	0.586		*PRMT8*
rs4759190	12	56119244	A	G	0.0048	$2.96X10^{-07}$	0.356		*CD63*
rs75564981	12	89939324	G	A	0.0102	$1.43X10^{-07}$	0.228	rs77029482; rs67661008; rs79105157; rs77685743; rs58302337; rs12580678; rs150210764	*POC1B*
rs73525667	13	81779870	G	A	0.0083	$3.89X10^{-07}$	0.243		*intergenic*
rs571544532	16	20921118	A	G	0.0014	$1.25X10^{-07}$	0.636		*LYRM1*

Supplemental Table 11: Sensitivity analysis for GWAS in those without baseline CVD: Lead SNPs, and other SNPs in the same loci, with GWAS significant associations with either troponin (at $p< 5X10^{-8}$)

Lead SNP	Chromosome	Position	Non-effect allele	Effect allele	Minor allele frequency	P-value	Beta	Other SNPs	Nearest gene
**Cardiac troponin I (n=18276)**									
rs542754544	1	37792490	G	T	0.0027	$3.97X10^{-08}$	-0.549		*Intergenic*
rs4253248	4	187155488	G	A	0.4881	$1.60X10^{-09}$	0.054	rs7661323; rs7683132; rs7377304; rs1053094; rs12331618; rs11132382; rs4862669; rs2048; rs4253238; rs1912826; rs3775298; rs1511801; rs4241815; rs4241816; rs4241817; rs4241818; rs4253252; rs3733402; rs4253254; rs66530140; rs35984397; rs4253281; rs4253282; rs1973612; rs2304595; rs4253304; rs4253305; rs4253311; rs2292423; rs4241822	*KLKB1, CYP4V2, F11*
rs71483973	10	75900464	A	G	0.1675	$1.69X10^{-09}$	0.076	rs10824069; rs2270552; rs2131959; rs3793921; rs2131956; rs6480718; rs6688; rs2395086; rs11817132; rs67594352	*VCL,AP3M1,ADK*
rs7481951	11	22271870	A	T	0.4101	$1.59X10^{-10}$	0.058	rs12790951; rs4635079; rs4244488; rs4244490; rs12807778; rs12290197; rs4275631; rs4478991; rs10833711; rs10833712; rs4408310; rs10741930; rs10833719; rs12574283	*ANO5*
rs61971809	14	22057505	T	C	0.0235	$2.25X10^{-08}$	-0.170	rs61971816	*OR10G1P*
rs148050755	17	62516959	A	C	0.0026	$5.64X10^{-11}$	-0.781		*CEP95,SMURF2*
rs2298711	18	57000469	T	A	0.1506	$1.54X10^{-11}$	0.085	rs117153087; rs12604758; rs41468947; rs17696641; rs12604424; rs12608430; rs149168942; rs77905572; rs4940867; rs78723480	*CPLX4,LMAN1*
**Cardiac Troponin T (n=18273)**									
rs16862512	1	169649691	T	C	0.0050	$2.70X10^{-08}$	0.364		*C1orf112*
rs190069901	4	159877305	A	T	0.0049	$3.87X10^{-08}$	0.373		*C4orf45*
rs75898208	4	186911043	G	A	0.0017	$1.64X10^{-10}$	0.799		*SORBS2*
rs184140292	9	8457116	T	C	0.0017	$2.66X10^{-08}$	0.703		*PTPRD*

Supplemental Figure 1: Unadjusted association of hsTnI and hsTnT, by approximate thirds, with the CVD outcome in those without baseline CVD (n=18,624). Each troponin was split into three groups (low; cTnT <3.0ng/L& cTnI ≤1.8ng/L, intermediate; cTnT 3.0-5.7ng/L & cTnI 1.9-3.0ng/L, high cTnT ≥5.8ng/L & cTnI ≥3.1ng/L), so that the proportion within each grouping was similar for each troponin. The solid line is the lowest Tn group (=1), the intermediate Tn group is the dashed line (=2), and the highest Tn group is the dotted line (=3).

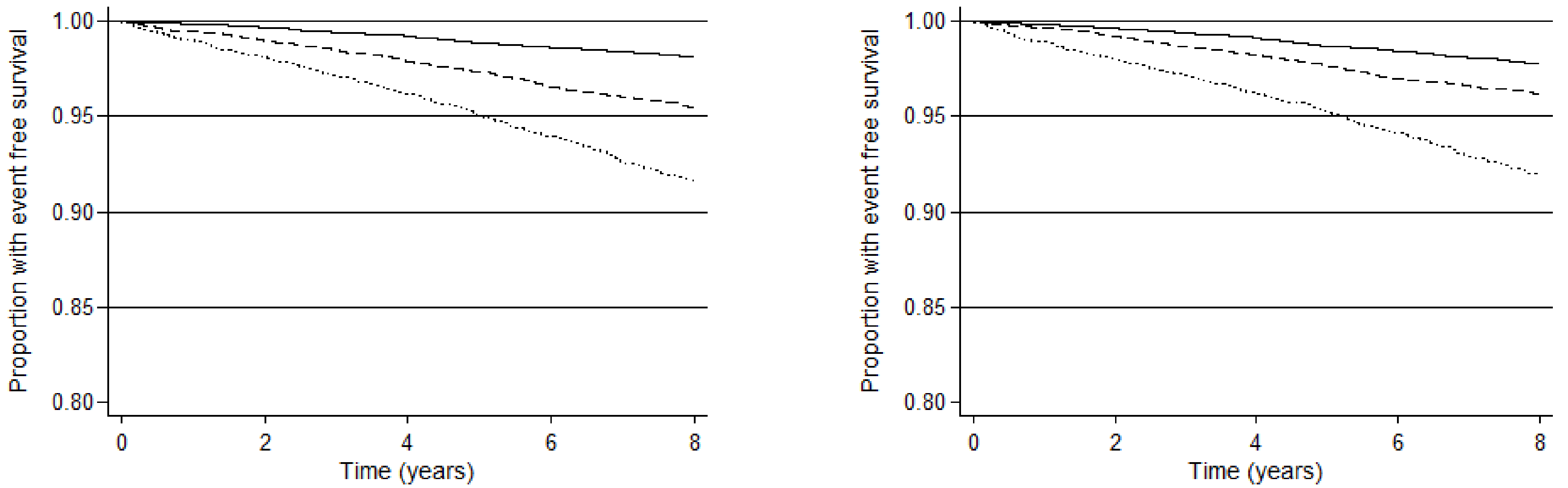

Number at risk

	0	2	4	6	8
hsTnI_tertile = 1	9242	9202	9152	9064	4900
hsTnI_tertile = 2	4843	4789	4720	4640	2026
hsTnI_tertile = 3	4539	4431	4314	4171	1781

Number at risk

	0	2	4	6	8
hsTnT_tertile = 1	8922	8883	8833	8746	4853
hsTnT_tertile = 2	4983	4938	4874	4794	1983
hsTnT_tertile = 3	4719	4601	4479	4335	1871

Supplemental Figure 2: Association of hsTnI and hsTnT unadjusted and adjusted (per table 2) with CVD death. The referent (HR=1) is undetectable levels of hsTnI and hsTnT respectively. Both x and y-axes on log scale.

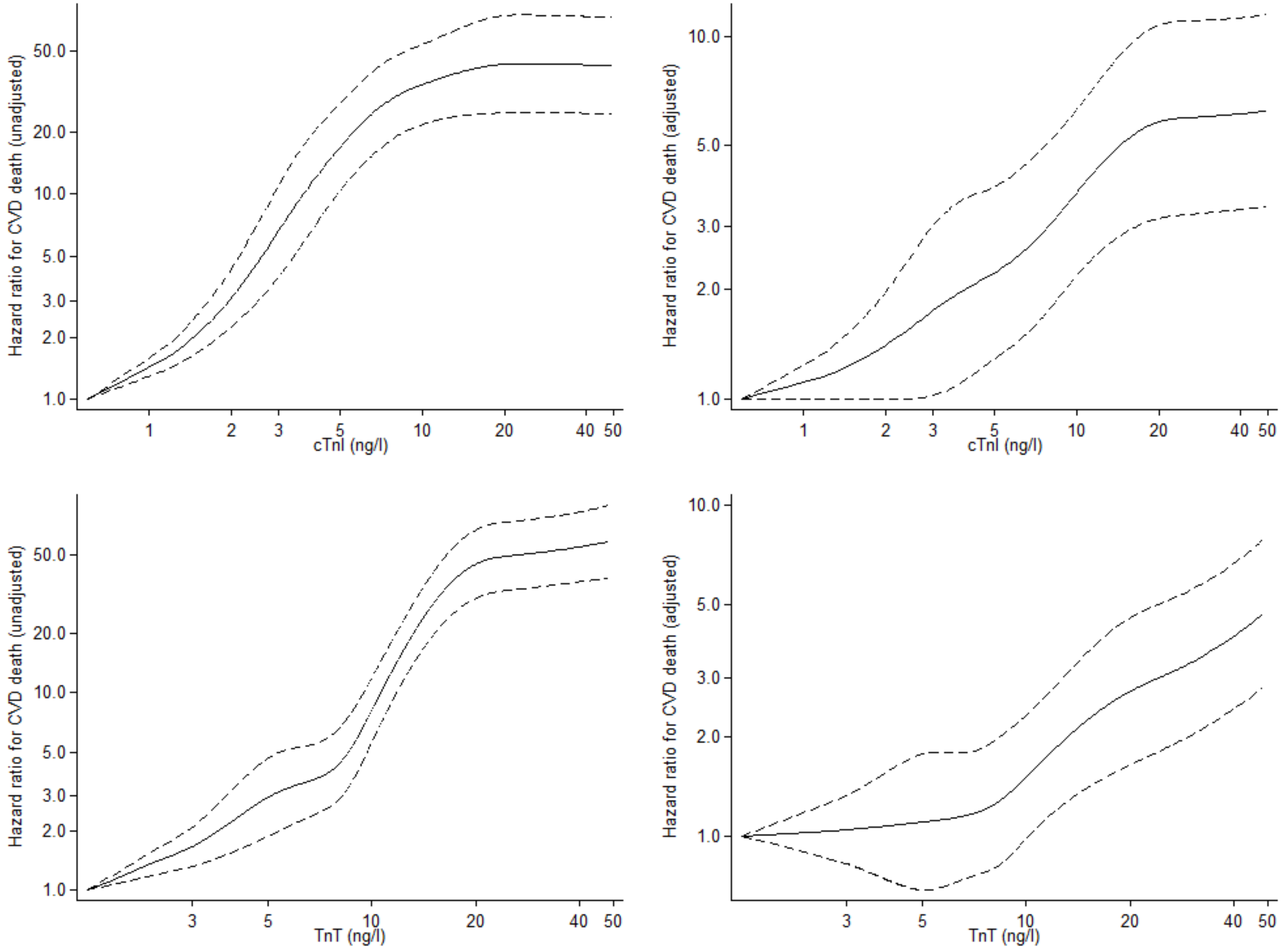

Supplemental Figure 3: Association of hsTnI and hsTnT unadjusted and adjusted (per table 2) with non-CVD death. The referent (HR=1) is undetectable levels of hsTnI and hsTnT respectively. Both x and y-axes on log scale.

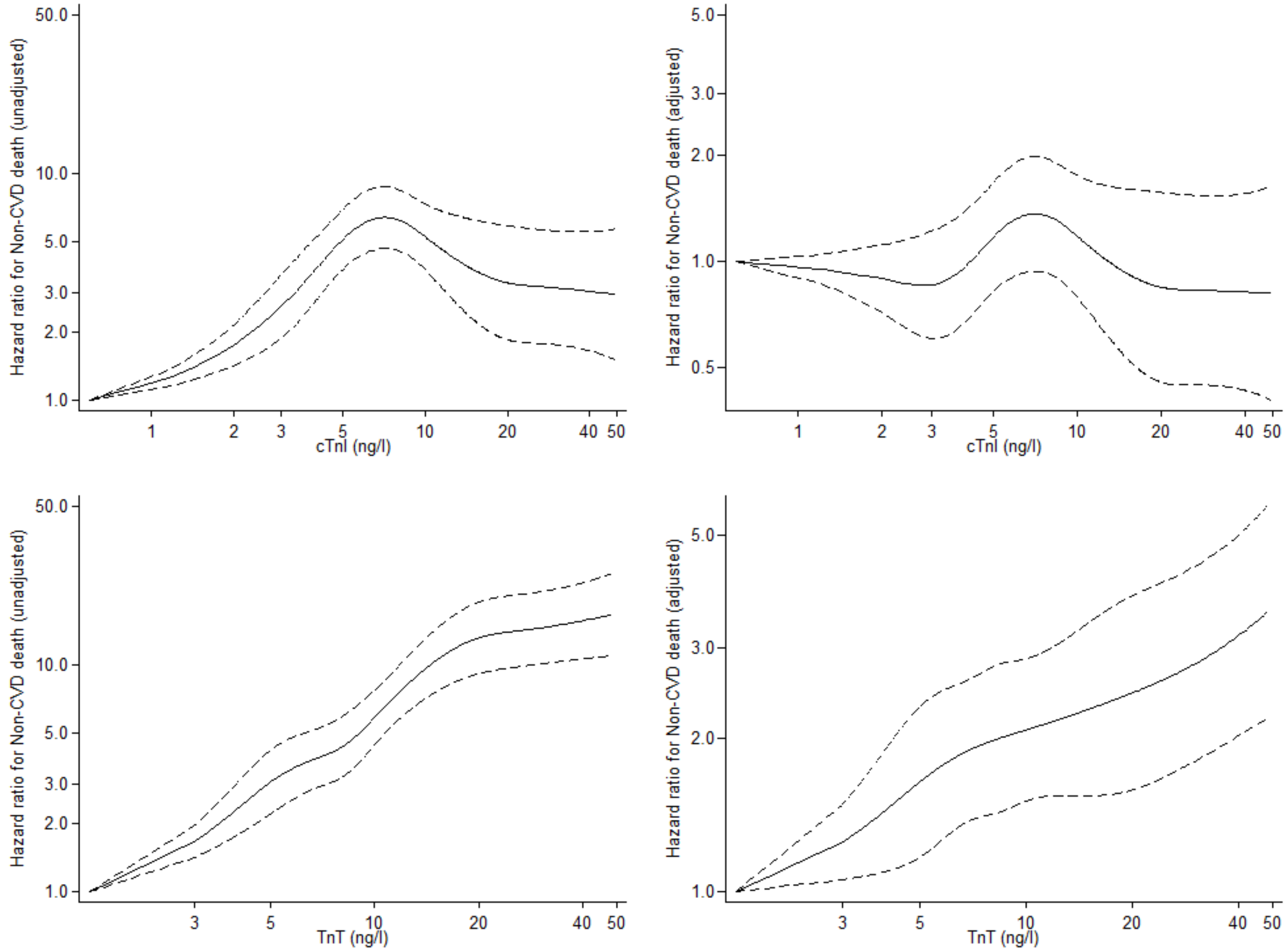

Supplemental Figure 4: Association of hsTnI and hsTnT unadjusted and adjusted (per table 2) with all cause mortality. The referent (HR=1) is undetectable levels of hsTnI and hsTnT respectively. Both x and y-axes on log scale.

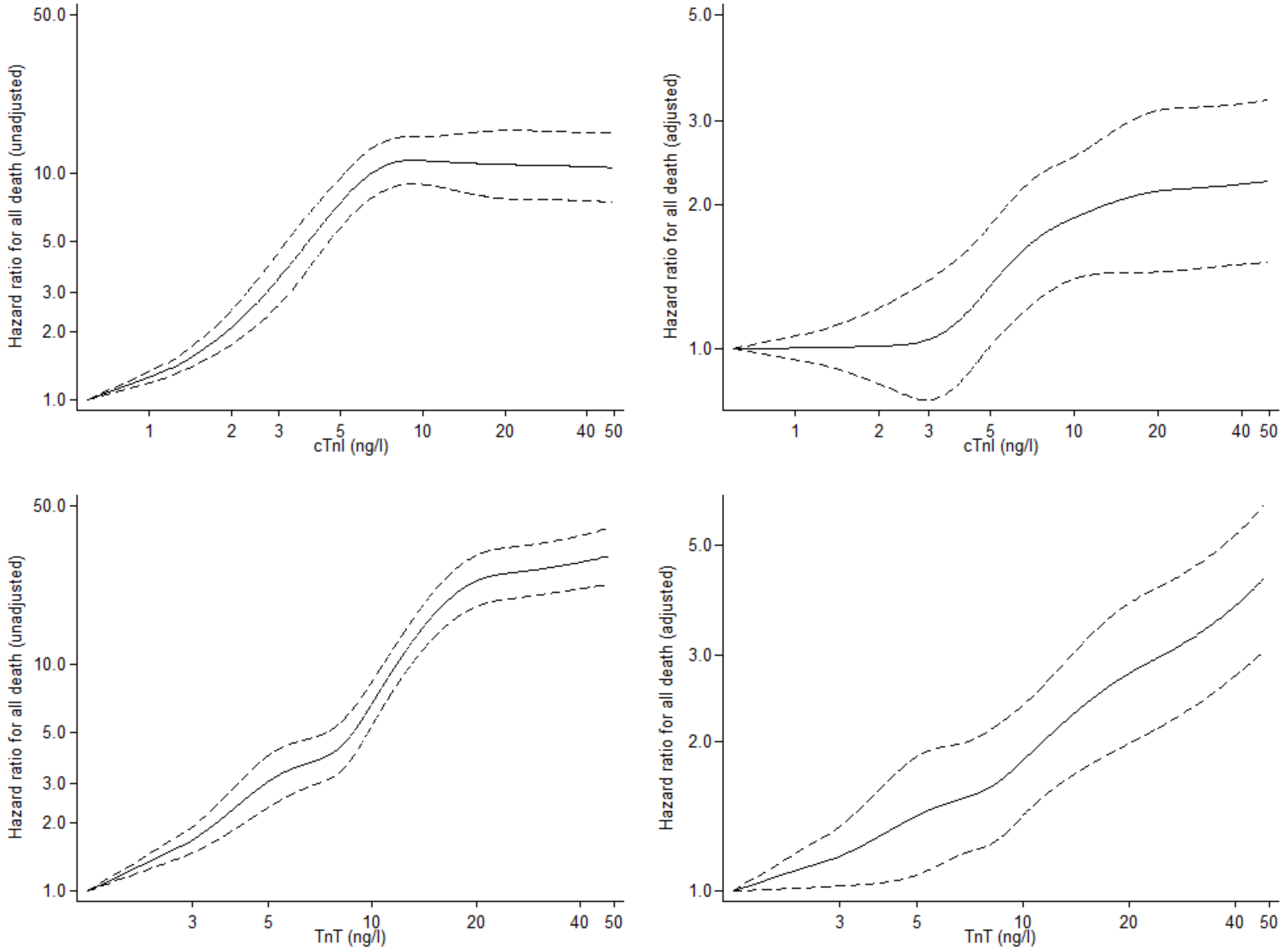

Supplemental Figure 5: Association of hsTnI and hsTnT unadjusted and adjusted (per table 2) with myocardial infarction. The referent (HR=1) is undetectable levels of hsTnI and hsTnT respectively. Both x and y-axes on log scale.

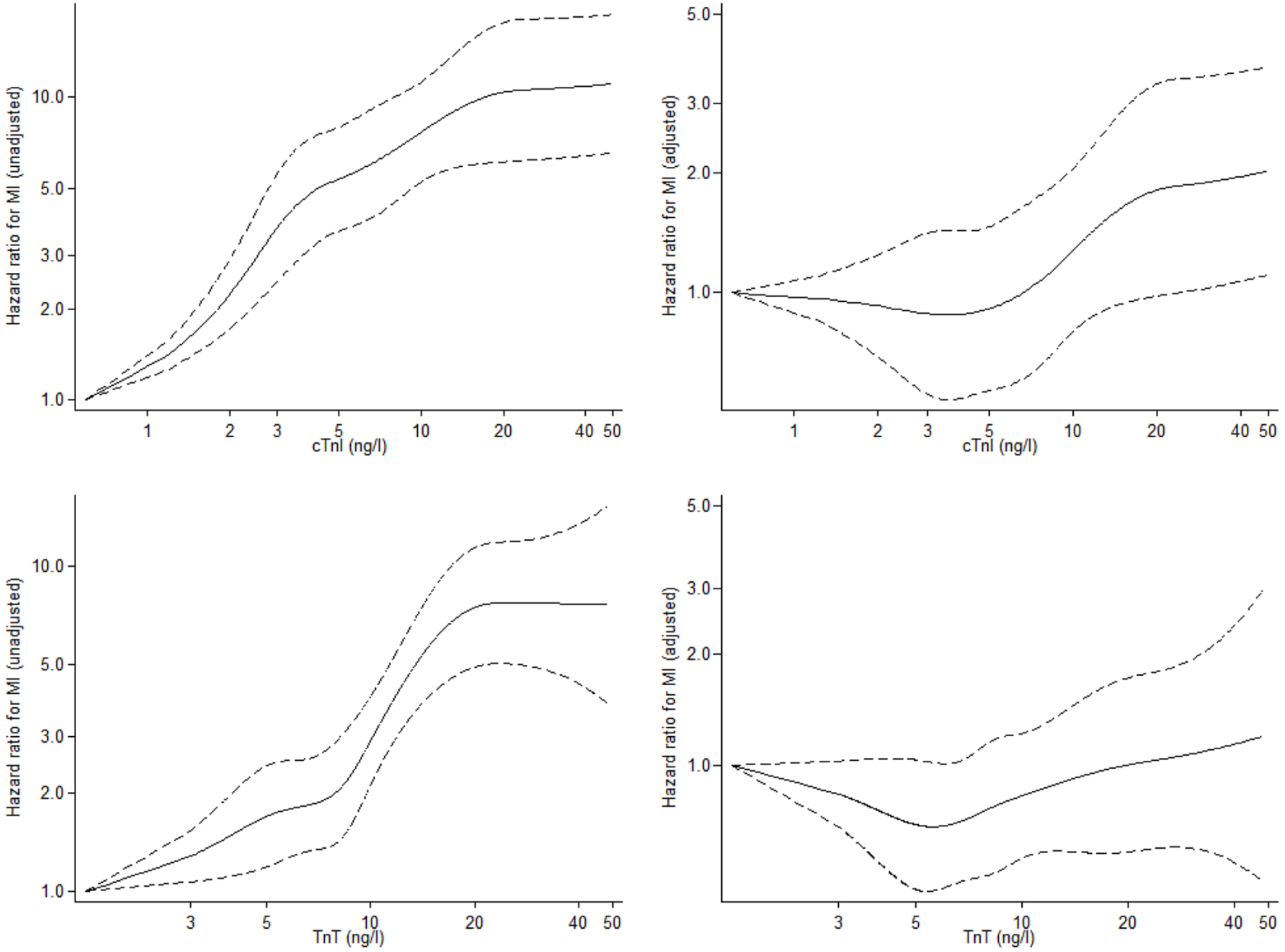

Supplemental Figure 6: Association of hsTnI and hsTnT unadjusted and adjusted (per table 2) with CHD. The referent (HR=1) is undetectable levels of hsTnI and hsTnT respectively. Both x and y-axes on log scale.

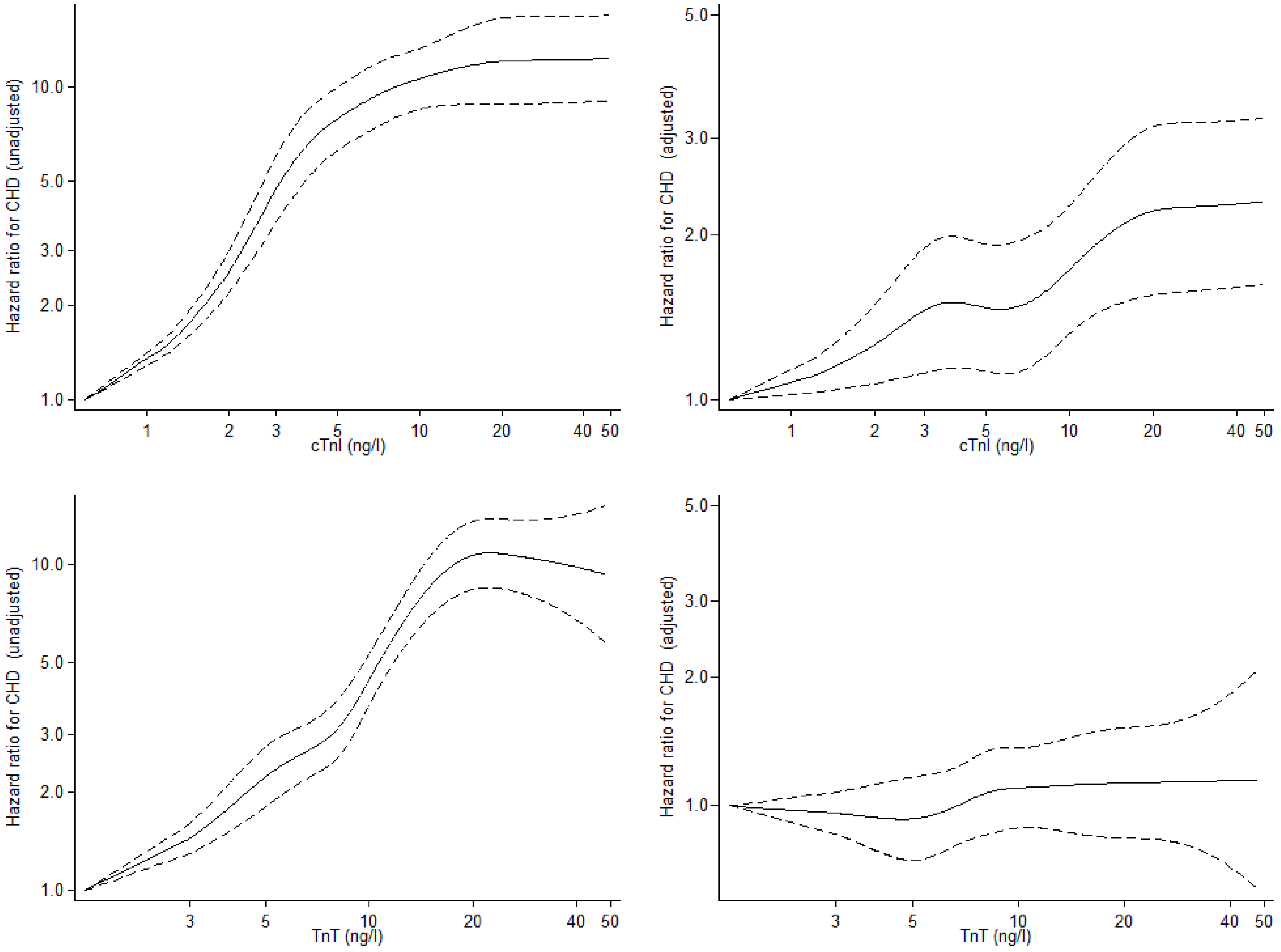

Supplemental Figure 7: Association of hsTnI and hsTnT unadjusted and adjusted (per table 2) with ischaemic stroke. The referent (HR=1) is undetectable levels of hsTnI and hsTnT respectively. Both x and y-axes on log scale.

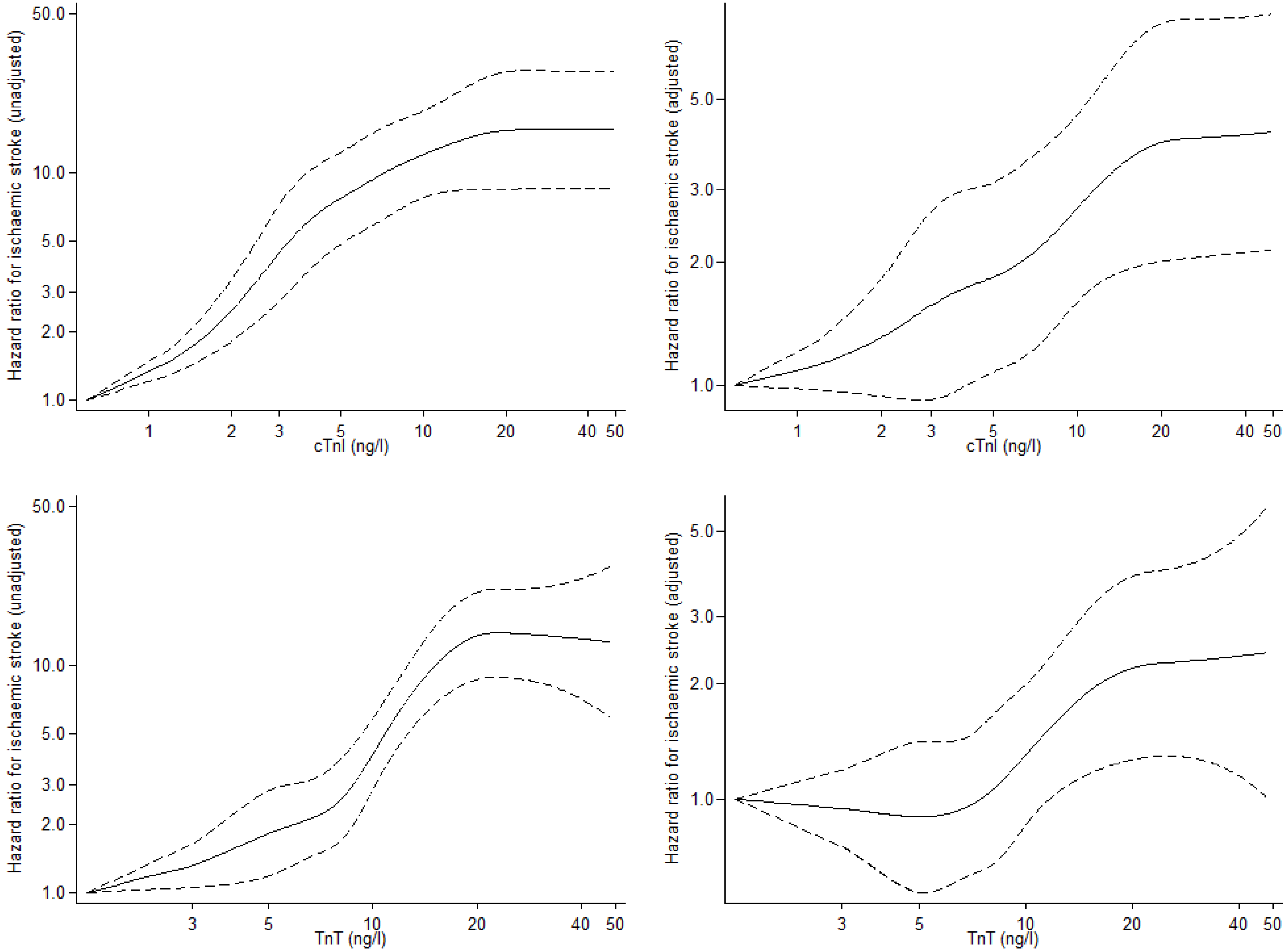

Supplemental Figure 8: Association of hsTnI and hsTnT unadjusted and adjusted (per table 2) with heart failure. The referent (HR=1) is undetectable levels of hsTnI and hsTnT respectively. Both x and y-axes on log scale

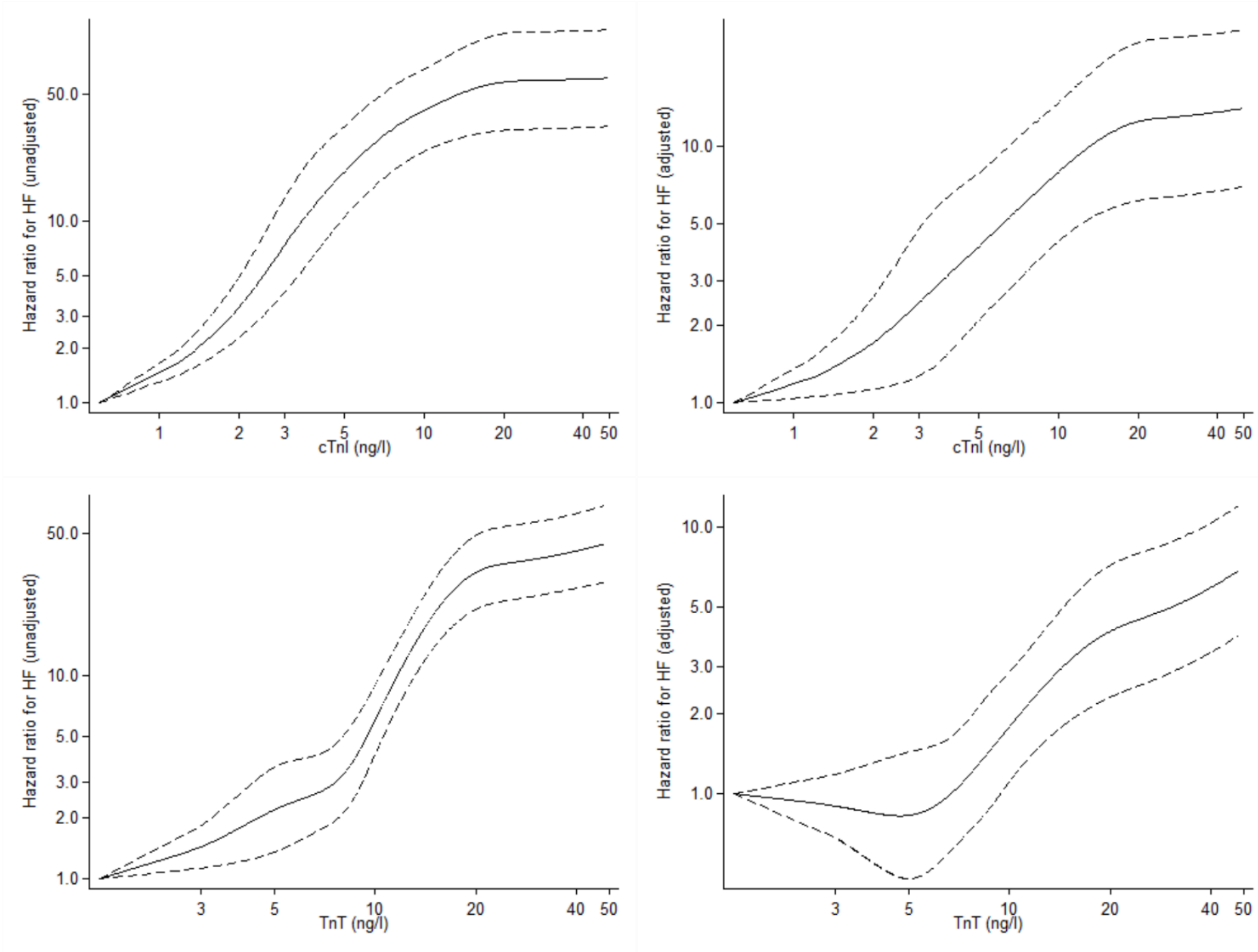

Supplemental Figure 9: Association of hsTnI and hsTnT unadjusted and adjusted (per table 2) with cancer. The referent (HR=1) is undetectable levels of hsTnI and hsTnT respectively. Both x and y-axes on log scale.

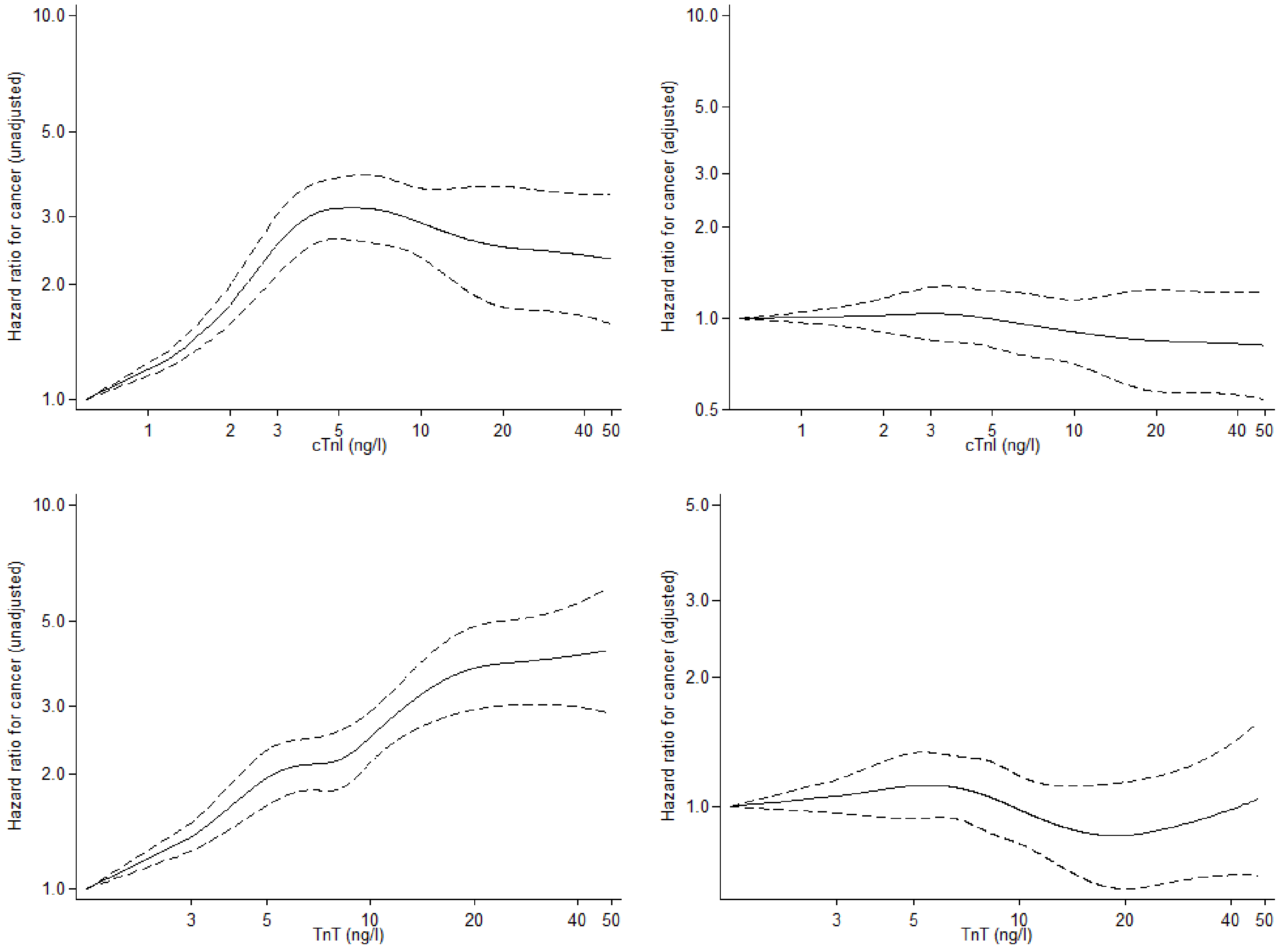

Supplemental Figure 10: Association of hsTnI (left) and hsTnT (right) with the composite CVD outcome, stratified by baseline risk factors, with p-value for interaction.

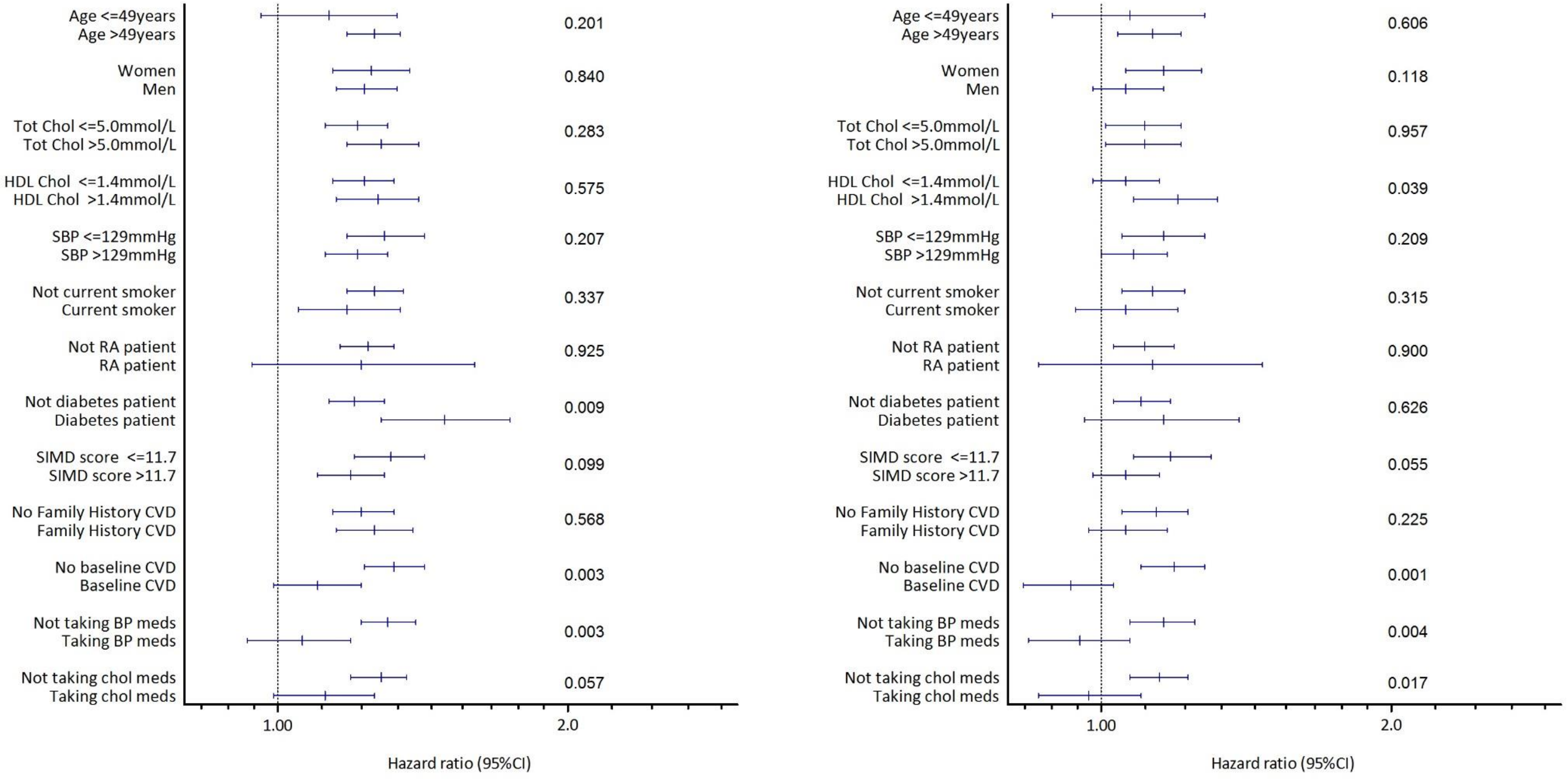

Supplement: Supplementary file 1 [file cir-139-2754-s001.pdf]
